# Integrating polygenic and methylation risk scores for pleural mesothelioma risk stratification

**DOI:** 10.1002/ijc.70316

**Published:** 2025-12-30

**Authors:** Khadija Sana Hafeez, Carla Debernardi, Alessandra Allione, Elton Jalis Herman, Simonetta Guarrera, Daniela Ferrante, Anna Aspesi, Marika Sculco, Marta La Vecchia, Carlotta Sacerdote, Federica Grosso, Christina M. Lill, Giovanna Masala, Marcela Guevara, Matthias B. Schulze, Salvatore Panico, Yaszan Asgari, Seehyun Park, Giovanna Tagliabue, Anne Tjønneland, Antonio Agudo, Elisabete Weiderpass, Corrado Magnani, Irma Dianzani, Paolo Vineis, Elisabetta Casalone, Giuseppe Matullo

**Affiliations:** ^1^ Department of Medical Sciences University of Turin Turin Italy; ^2^ Italian Institute for Genomic Medicine, IIGM Turin Italy; ^3^ Candiolo Cancer Institute FPO, FPO‐IRCCS Candiolo Italy; ^4^ Department of Translational Medicine, Unit of Medical Statistics and Cancer Epidemiology University of Eastern Piedmont, CPO‐Piedmont Novara Italy; ^5^ Department of Health Sciences Università del Piemonte Orientale Novara Italy; ^6^ Epidemiology Unit, Local Health Authority Novara Novara Italy; ^7^ Mesothelioma Unit, AO SS. Antonio e Biagio e Cesare Arrigo Alessandria Italy; ^8^ Ageing Epidemiology Research Unit, School of Public Health Imperial College London UK; ^9^ Institute of Epidemiology and Social Medicine, University of Münster Münster Germany; ^10^ Clinical Epidemiology Unit, Institute for Cancer Research Prevention and Clinical Network (Ispro) Florence Italy; ^11^ Navarra Institute of Public and Labor Health Pamplona Spain; ^12^ Center for Biomedical Research in Epidemiology and Public Health (Ciberesp) Madrid Spain; ^13^ Navarra Institute for Health Research (Idisna) Pamplona Spain; ^14^ Department of Molecular Epidemiology German Institute of Human Nutrition Potsdam‐Rehbruecke Nuthetal Germany; ^15^ Institute of Nutritional Science, University of Potsdam Nuthetal Germany; ^16^ Federico Ii University Naples Italy; ^17^ Paris‐Saclay University, Uvsq, Inserm, Gustave Roussy, Cesp Villejuif France; ^18^ Cancer Registry Unit, IRCCS Foundation National Cancer Institute of Milan Milan Italy; ^19^ Danish Cancer Institute Copenhagen Denmark; ^20^ Unit of Nutrition and Cancer Catalan Institute of Oncology, ICO L'Hospitalet de Llobregat Spain; ^21^ Nutrition and Cancer Group, Epidemiology, Public Health, Cancer Prevention and Palliative Care Program Bellvitge Biomedical Research Institute, IDIBELL L'Hospitalet de Llobregat Spain; ^22^ International Agency for Research on Cancer World Health Organization Lyon France; ^23^ Department of Epidemiology and Biostatistics, School of Public Health, MCR Centre for Environment and Health Imperial College London London UK; ^24^ Medical Genetic Service AOU Città della Salute e della Scienza Turin Italy

**Keywords:** genome‐wide association analyses (GWAS), methylation risk score (MRS), pleural mesothelioma (PM), polygenic risk score (PRS), risk assessment

## Abstract

Pleural mesothelioma (PM) is a lethal cancer primarily caused by asbestos exposure. Not all exposed individuals develop PM, suggesting the involvement of additional factors. This underscores the need for robust predictive models integrating biomarkers from multi‐omic domains to improve risk stratification and early detection. We developed and evaluated polygenic risk scores (PRS) and methylation risk scores (MRS) using a retrospective case–control study (749 participants: 387 PM cases, 362 controls) and a nested case–control European Prospective Investigation into Cancer and Nutrition (EPIC)‐Meso study (268 participants: 134 preclinical PM cases, 134 matched controls) within the EPIC cohort. Genome‐wide association analyses in the retrospective case–control study identified PM‐associated variants. The PRS (1123 SNPs with *p* < 0.001) in the retrospective training subset stratified disease risk in the test set (ORs 3.46–9.54 across top percentiles) and improved model discrimination (AUC = 0.75 vs. 0.71 in baseline model, *p* = 0.04). In EPIC‐Meso, PRS performance was limited (AUC = 0.52). External validation in the UK‐Biobank (UKBB) confirmed a modest but consistent association with PM‐risk. A Meta‐PRS derived from the UKBB‐FinnGen meta‐analysis replicated this trend in the full retrospective dataset, showing higher OR across top percentiles (2.5–12.3) and improved discrimination (AUC 0.74 vs. 0.72, *p* = 0.016). MRS, with 68 differentially methylated CpGs (effect‐size >|0.10|, FDR *p* < 0.05) in the retrospective training set, increased the AUC from 0.66 to 0.85 (*p* < 0.001) in the test set and from 0.51 to 0.62 in EPIC‐Meso. PRS was most predictive in low‐exposure groups, while MRS remained robust across exposure levels. Combined PRS‐MRS models improved discrimination. Integrating multi‐omic biomarkers can enhance PM‐risk stratification and support earlier, targeted interventions in high‐risk asbestos‐exposed groups.

## Graphical abstract

What's new?

Asbestos exposure is a major risk factor for pleural mesothelioma (PM). Most asbestos‐exposed individuals do not develop PM, suggesting that it arises from a complex interplay between environmental and genetic factors. This study examined the utility of polygenic risk scores (PRS) and methylation risk scores (MRS) in incorporating genetic susceptibility into PM risk prediction. While MRS outperformed PRS across exposure levels, combined PRS and MRS improved risk stratification, especially among individuals with low asbestos exposure, where genetic predisposition may be more informative. PRS–MRS integration may improve early detection and guide personalized surveillance for asbestos‐exposed individuals at high PM risk.

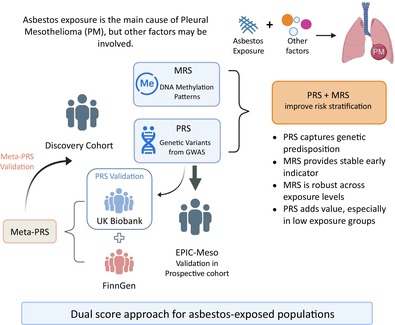

**Abbreviations**
AUC: area under the curve
CI: confidence interval
CpG: cytosine‐phosphate‐guanine dinucleotide
DNAm: DNA methylation
EPIC: European Prospective Investigation into Cancer and Nutrition
EPIC‐Meso: EPIC‐mesothelioma substudy
FDR: false discovery rate
GWAS: Genome‐Wide Association Study
JEM: job exposure matrix
KEGG: Kyoto Encyclopedia of Genes and Genomes
MRS: methylation risk score
NRI: net reclassification index
OR: odds ratio
PCA: principal component analysis
PM: pleural mesothelioma
PRS: polygenic risk score
SNP: single nucleotide polymorphism

## 1 INTRODUCTION

Pleural mesothelioma (PM) is a rare and aggressive cancer, which originates in the pleura and is linked to asbestos exposure. Its poor prognosis and long latency period underscore the need for early identification of at‐risk individuals to improve outcomes. However, early diagnosis remains challenging due to the absence of reliable, noninvasive biomarkers.

While various candidates have been explored, including circulating proteins (e.g., mesothelin, calretinin, fibulin), peripheral blood DNA methylation (DNAm) variation, and microRNAs (miRNAs)^1^, ^2^, ^3^, ^4^, ^5^ as potential biomarkers, translating these into clinical practice remains limited.

In this context, polygenic risk scores (PRS) and methylation risk scores (MRS) have emerged as promising tools in molecular epidemiology, providing quantitative assessments of an individual's genetic and epigenetic contribution to diseases.^6^, ^7^, ^8^, ^9^, ^10^ PRS estimates risk by aggregating the effects of multiple genetic variants, each contributing a small risk. Similarly, MRS integrates the influence of DNAm patterns across various loci, reflecting both inherited and environmental influences to capture multifactorial information about underlying risk that leads to clinical conditions.^11^

This study explores the potential of PRS and MRS to improve risk stratification for PM in asbestos‐exposed individuals. We hypothesize that an integrated model can provide a more accurate risk assessment than either score alone, ultimately enhancing risk stratification and enabling tailored approaches for early detection and intervention.

## 2 MATERIALS AND METHODS

### 2.1 Study cohorts

The retrospective case–control study consisted of 820 Italian participants of European ancestry enrolled from registry‐based population and hospital‐based case–control studies conducted in the asbestos‐exposed areas of Piedmont and Liguria as previously described.^12^ Genetic ancestry was verified using principal component analysis (PCA). Cases were histologically confirmed and recruited from hospitals or cancer registries, and controls were age‐ and sex‐matched individuals from population rosters, hospitals, or volunteer groups. Across all panels, blood samples were collected prior to treatment and processed for DNA extraction. Asbestos exposure was evaluated by an expert industrial hygienist based on detailed occupational histories and categorized as “no/unlikely,” “low,” or “high.” For the DNAm analysis, a subset of 300 participants (163 PM cases, 137 controls) were included. Inclusion in the methylation study required: (i) availability of high‐quality DNA, (ii) documented asbestos exposure level, and (iii) exposure above background, as defined previously.^13^

The EPIC‐Meso cohort was nested within the EPIC study, which enrolled 521,468 healthy individuals of predominantly European ancestry across 10 European countries between 1993 and 1998.^14^ Within EPIC, 134 participants developed PM after an average follow‐up of 8.3 years (Table S1). These participants and the matched disease‐free controls, 1:1 by age (±1.5 years), sex, country of recruitment, and level of asbestos exposure, were previously described in ref. 1. Asbestos exposure was estimated using a semi‐quantitative job‐exposure matrix, JEM^2^ and treated as a categorical variable: (0 = no; 1 = low; 2 = high).

In the UK Biobank (UKBB),^15^ we analyzed 547 individuals with mesothelioma (ICD‐10:C45) (accessed 31 March 2025), including 449 with PM (C45.0) and 430,984 controls without relevant ICD‐10 codes. No ancestry‐based filtering was applied to maximize the population diversity.

In a subsequent analysis performed after the latest UKBB data release (accessed on 27 June 2025), the number of cases had increased, with 511 mesothelioma cases (C45.0/C45.1/C45.2) and 487,409 controls available. Summary statistics from the Finnish dataset were obtained from the FinnGen R12 release,^16^ comprising 442 cases and 378,749 controls.

### 2.2 Genotyping and data preprocessing

In the retrospective case–control study, 740 participants were genotyped on the HumanCNV370‐Quad BeadChip (Illumina), and 80 on the Human610‐Quad array. EPIC‐Meso participants were genotyped using the Infinium Global Screening Array‐24 v3.0/OmniExpress. Genotype calling was performed using GenomeStudio (Illumina Inc., San Diego, CA, USA). The quality‐control (QC) steps are detailed in (Table S2). Following stringent QC, 23 related individuals (IBD >0.125), 8 heterozygosity outliers (>3 SD from the mean), and 13 population stratification outliers, detected via iterative PCA, in PLINK v1.9 (RRID:SCR_001757)^17^ were excluded from the retrospective case–control study. Samples with missing information (*n* = 8) were excluded from the analysis. The EPIC‐Meso dataset was filtered using the same criteria. After filtering, 749 individuals (387 cases and 362 controls) remained in the retrospective case–control study and 264 (133 cases and 131 controls) in the EPIC‐Meso cohort.

### 2.3 Imputation and association testing

Imputation was performed using the Michigan Imputation Server on the HRC r1.1 2016 (hg19) panel^18^, ^19^ based on the European population. We imputed the two quality‐controlled subsets of the main retrospective case–control study individually and merged them. Indels were normalized, multiallelic sites were split into biallelic records, and only variants with a high imputation quality (INFO score >0.8) were retained.

Single‐variant association analyses were conducted using SAIGE (v1.1.6.3) in R (v3.6.3),^20^ adjusting for age, sex, asbestos‐exposure, and population stratification, with the number of principal components determined from genomic inflation factors (three PCs for the retrospective dataset, λ = 1.018; four for EPIC‐Meso, λ = 1.059). For construction of the SAIGE null model, variants with MAF ≥0.001 were included to ensure stable relationship matrix estimation, while association testing was restricted to variants with MAF ≥0.01 and minor allele count (MAC) ≥20. The same MAF filters were applied to the EPIC‐Meso dataset. The results were meta‐analyzed based on the fixed‐effect inverse‐variance model using METAL v2011‐03‐25 (RRID:SCR_002013).^21^ Annotation was performed using Ensembl‐VEP (RRID:SCR_002344)^22^ and SNPnexus (RRID:SCR_005192),^23^ and pathway enrichment analyses were conducted using ShinyGo, KEGG (RRID:SCR_012773), EnrichR (RRID:SCR_001575), and GSEA (MSigDB).^24^, ^25^, ^26^, ^27^ Functional mapping and annotation were further explored using FUMA^28^ and GRASP^29^ was used to explore genotype–phenotype links across various GWAS.

Genotyping and imputation of the UKBB participants followed standardized protocols as described in the UKBB documentation.^15^

Interaction effects between GWAS‐significant SNPs and asbestos exposure were assessed using logistic regression models. Full details of the methodology and results are provided in Data S1.

### 2.4 DNA methylation analysis

DNAm levels were measured in whole blood. In the retrospective case–control study, 300 DNA participants (163 PM cases; 137 controls) were analyzed using the Infinium HumanMethylation450 BeadChip (Illumina, San Diego, CA) and in the EPIC‐Meso cohort, 268 participants (134 PM cases and 134 matched controls) were analyzed with the Infinium Methylation EPIC BeadChip. The protocols for DNA extraction, BeadChip processing, and data QC were described previously.^1^, ^13^ β‐values were used for all analyses to preserve interpretability and data were quantile‐normalized to minimize platform‐specific bias. The retrospective dataset was split 50:50 into training and test sets; differential methylation was assessed in the training set using multiple regression (R's glm), adjusting for age, sex, asbestos exposure, batch, BeadChip controls, and the first two genetic PCs. Estimated white blood cell proportions were derived but not included as a covariate, consistent with our previous work, as asbestos exposure and mesothelioma can alter immune‐cell composition; adjusting for these shifts could remove biologically relevant signal rather than confounding.^13^

### 2.5 Risk scores construction

Risk scores were calculated as the weighted sum of preselected features using an in‐house pipeline. For PRS, the retrospective case–control study was randomly split 50:50 into training (*n* = 374; 193 cases, 181 controls) and test sets (*n* = 375; 194 cases, 181 controls, with 6,151,859 SNPs post‐QC). PRS were generated at multiple inclusion thresholds (5 × 10^−8^ to 1 × 10^−3^) following LD clumping (*r* ^2^ < 0.1 within 250 kb). The model with the highest AUC and most significant Kolmogorov–Smirnov (K–S) statistic was retained and thus variants with *p* < 0.001 in the training set were selected and used to compute the PRS on the test set. The PRS derived in the retrospective case–control study was validated in the UKBB dataset (ICD‐10:C45; accessed: 31 March 2025). To account for LD patterns or possible allele frequency heterogeneity, scores in EPIC‐Meso were normalized using a PCA‐based method with pgsc_calc v2.0.0‐alpha.4.^30^

To further assess robustness and minimize potential overfitting from the smaller training GWAS, we additionally constructed a Meta‐PRS using independent summary statistics from the UKBB‐FinnGen mesothelioma meta‐analysis (~953 cases, 866,158 controls). Meta‐analysis was performed using GWAMA.^31^ The Meta‐PRS was evaluated in the entire retrospective case–control study (*n* = 749). Variants were harmonized, excluding palindromic and large‐effect sites (|β| >0.5) to reduce the influence of potentially unstable effect estimates. PRS were computed at multiple inclusion thresholds after LD clumping, and the best‐performing model (*p* < 1 × 10^−4^; 93 variants) was used for additional validation.

The MRS was derived by splitting the 300‐sample retrospective DNAm dataset into equal training (67 controls, 83 cases) and test‐sets (70 controls, 80 cases) containing 346,441 CpGs measured as β‐values. CpGs with an effect size >|0.10| and FDR *p* < 0.05 from differential methylation analysis on the training set were used to calculate the MRS. The scores were validated in the EPIC‐Meso study.

### 2.6 Combined PRS‐MRS analysis

We evaluated four logistic regression models incorporating PRS, MRS, and their combination to differentiate cases from controls in both retrospective and EPIC‐Meso cohorts. Model performance was assessed using the area under the curve (AUC). The sensitivity and specificity were calculated for participants with high risk scores (above the 80th percentile).

### 2.7 Statistical analysis

Cohort comparisons used Fisher's exact and *t*‐tests. Analyses were conducted in R (v3.6.3) using glm for regression, pROC for AUC estimation, and ggplot2 (RRID:SCR_014601) for visualization, with significance set at *p* < 0.05.

## 3 RESULTS

The characteristics of the participants in the two cohorts are summarized in Table 1. After QC, the retrospective case–control study included 749 individuals (387 PM cases, 362 controls) with 7,901,261 variants, while the EPIC‐Meso cohort included 264 individuals (133 cases, 131 controls) with 9,254,605 variants. Sex distribution did not differ between cohorts (Fisher's *p* = 0.60 for males, *p* = 1.0 for females), but participants in the retrospective case–control study were older (*p* < 0.0001). Asbestos‐exposure also differed significantly (Fisher's *p* < 0.0001), with notable differences in the “no exposure” (*p* = 7.5 × 10^−6^) and “high exposure” (*p* = 0.012) categories. PCA showed two clusters reflecting Northern and Southern Italian ancestry in the retrospective data and a homogeneous European profile in EPIC‐Meso. Manhattan and QQ plots confirmed well‐controlled genomic inflation in both datasets (Figures S1–S3).

**TABLE 1:** Sample sizes and demographics of the mesothelioma retrospective cohort and the EPIC‐Meso prospective cohort.

	Retrospective study		EPIC‐Meso study	
	Cases, *N* (%)	Cancer free controls, *N* (%)	Pre‐clinical cases, *N* (%)	Cancer free controls, *N* (%)
*N* (%*)	387 (51.67)	362 (48.33)	134 (50)	134 (50)
Sex (%**)				
Males	277 (71.58)	254 (70.17)	107 (79.85)	107 (79.85)
Females	110 (28.42)	108 (29.83)	27 (20.15)	27 (20.15)
Age (mean, SD)	68.19, ±10.18	63.51, ±11.61	57.14, ±6.94	57.30, ±7.05
Males	67.99, ±10.12	63.39, ±11.42	57.53, ±7.05	57.52, ±7.05
Females	68.71, ±10.37	63.31, ±12.10	56.42. ±7.13	56.42, ±7.11
Asbestos exposure (%**)				
No exposure	17 (4.39)	107 (29.56)	21 (15.67)	21 (15.67)
Low exposure	153 (39.53)	153 (42.27)	28 (20.90)	28 (20.90)
High exposure	192 (49.61)	102 (28.18)	43 (32.09)	43 (32.09)
NA	25 (6.46)	0	42 (31.34)	42 (31.34)

*Note*: Percentages were calculated relative to the entire subgroup (*) or the number of individuals (*N*) in each column (**). “NA” indicates individuals for whom exposure data was unavailable. Statistical comparisons between the cohorts were made using Fisher's exact test and *t* test.

### 3.1 Genome wide association analysis

In the retrospective case–control study, 17 SNPs achieved genome‐wide significance (*p* < 5 × 10^−8^; Tables 2 and S3), mapping to five loci, including genes implicated in PM and cancer biology such as *OAT*, *ITGBL1*, *PLA2R1*, and *MICAL3*. Additional variants in *ATG5*, *THRB*, and *PVT1* showed suggestive significance (*p* < 1 × 10^−5^), consistent with previous findings.^12^

**TABLE 2:** Top variants from the retrospective GWAS with mapped genes.

SNP ID	Chr location	POS	Ref allele	Alt allele	Overlapped gene	Nearest upstream gene	Nearest downstream gene	*p*‐Value
rs1079859	16.q12.1	51874423	C	A	‐	RP11‐7O14.1	C16orf97	4.32E‐15
rs2122342	8.q21.13	83955796	A	C	‐	CTD‐2272D18.2	RP11‐296C13.1	3.11E‐14
rs12912424	15.q26.3	101319296	A	T	RP11‐66B24.5	‐	‐	4.39E‐14
rs2459219	10.q26.13	126057825	G	T	‐	CHST15	OAT	1.03E‐13
rs12822999	12.p13.31	9968349	T	G	‐	CD69	RP11‐75L1.6	1.40E‐12
rs9539946	13.q21.31	64718463	T	G	‐	LINC00355	LGMNP1	2.03E‐12
rs12900352	15.q12	27816841	G	A	‐	RP11‐100M12.3	RP11‐30G8.2	2.45E‐12
rs1351034	15.q12	27923343	T	C	‐	RP11‐30G8.2	RP11‐30G8.1	7.08E‐12
rs1469849	13.q33.1	102299158	G	C	ITGBL1	‐	‐	9.43E‐12
rs1877196	2.q24.2	160915150	G	T	PLA2R1	‐	‐	2.28E‐10
rs401224	22.q11.21	18280799	T	A	MICAL3	‐	‐	4.32E‐09
rs113946246	13.q11	19429318	T	C	ANKRD20A9P	‐	‐	2.54E‐08
rs2490010	13.q11	19442530	A	G	ANKRD20A9P	‐	‐	3.62E‐08
rs67206049	13.q11	19429058	C	T	ANKRD20A9P	‐	‐	4.18E−08
rs2876969	7.p12.1	51676796	A	C	‐	ROBO2P1	RP4‐718N17.2	4.18E‐08
rs2440012	13.q11	19440123	C	G	ANKRD20A9P	‐	‐	4.60E‐08
rs1838126	13.q11	19444828	C	T	ANKRD20A9P	‐	‐	4.96E‐08

Abbreviations: Chr Location, cytogenetic band region; POS, base pair position based on the Genome Reference Consortium Human Build 37 (Hg19); Ref Allele, reference allele; Alt allele, effect/alternate allele; Nearest Upstream/Downstream gene: the nearest protein coding gene within 100 kb from the lead SNP (if any); *p*‐value, GWAS *p*‐value.

In the EPIC‐Meso, no SNPs met genome‐wide significance, though 30 variants reached suggestive significance (*p* < 1 × 10^−5^; Table S4). Meta‐analysis combining both cohorts identified six SNPs with suggestive significance, four with consistent effect sizes and directions (*I* ^2^ = 0%) (Table 3) and two showing moderate heterogeneity (rs2122342, *I* ^2^ = 79%; rs742109, *I* ^2^ = 64%) Figure S4. Three variants on chromosome 7q35‐37 (rs11980724, rs11973750, and rs17170347) linked to *TPK1* were in strong LD.

**TABLE 3:** Top suggestive variants from meta‐analysis of retrospective and EPIC‐Meso GWAS.

SNP ID	Ref allele	Alt allele	Chr location	OR (95%CI)	*p*‐Value	Annotated gene	Consequence	HetISq	HetPVal
rs2122342	A	C	8.q21.13	0.41 (0.33–0.50)	3.65E−13	‐	‐	78.6	0.03
rs742109	T	C	6.q21	1.61 (1.31–1.98)	3.60E−06	‐	‐	64.1	0.10
rs11980724	C	T	7.q35	0.36 (0.24–0.56)	5.39E−06	TPK1	Intronic	0	0.36
rs11973750	G	A	7.q36	0.36 (0.24–0.56)	5.86E−06	TPK1	Intronic	0	0.37
rs17170347	C	T	7.q37	0.36 (0.24–0.56)	5.86E−06	TPK1	Intronic	0	0.37
rs11939676	A	G	4.p15.1	0.45 (0.32–0.64)	9.45E−06	PCDH7	Intronic	0	0.52

Abbreviations: Alt Allele, effect/alternate allele; annotated gene, overlapping gene; consequence, gene consequences; Chr location, cytogenetic band region; HetISq, Heterogeneity index I2 by Higgins et al. 2003; OR (95%CI), overall odds ratio and 95% confidence interval for meta‐analysis; *p*‐value, meta‐analysis *p*‐value; Ref allele, reference allele; HetPVal, *p*‐value for the heterogeneity test.

### 3.2 Pathway enrichment

Over‐representation analysis of 11 overlapping or neighboring genes genome‐wide significantly associated in the retrospective case–control study (Table 2) revealed individual associations with important pathways, although no single pathway was shared across this restricted gene set. For example, *CHST15* was linked to the glycosaminoglycan biosynthesis pathway (FDR = 0.026), *OAT* to the arginine and proline metabolism pathway (FDR = 0.033), and *PLA2R1* to both the phagosome (FDR = 0.058) and tuberculosis (FDR = 0.058) pathways (Tables S5, S6, Figure S5).

Meta‐analysis derived variants (*p* < 1 × 10^−5^) were significantly enriched in pathways related to thiamine metabolism, particularly involving *TPK1* and *SLC25A19* (Table S7), indicating a potential role for energy metabolism and mitochondrial function in PM pathogenesis. The FUMA results for the meta‐analysis are detailed in Table S8A, B, Figure S6.

### 3.3 Differential methylation analysis and enrichment analysis

Sixty‐eight CpGs showed significant differential methylation between cases and controls (|effect‐size| >0.10, FDR <0.05) in the training set of the retrospective case–control study, including 58 hypomethylated and 10 hypermethylated sites, mapped to 54 genes (Table S9). The associated genes were primarily involved in lipid and monocarboxylic acid metabolism, cellular catabolic processes, and overlapped with MSigDB gene sets linked to miRNA regulation, DNA repair, and inflammation, processes known to contribute to PM development.

### 3.4 Risk scores

**FIGURE 1:**
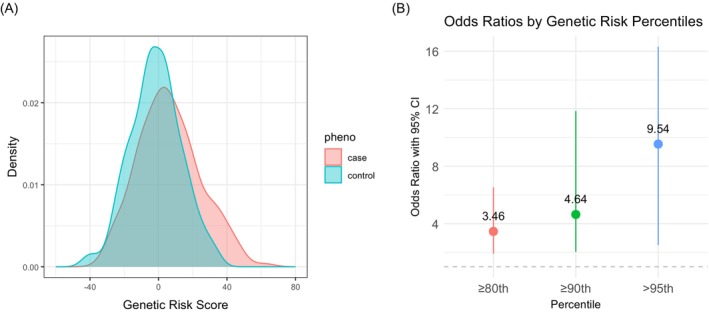
Polygenic risk score (PRS) distribution and risk stratification in the retrospective case–control study. (A) Density plot showing the distribution of PRS for mesothelioma cases (red) and controls (blue). The X‐axis represents PRS values, and the Y‐axis indicates the density. The curves illustrate the relative frequency of PRS values in each group. (B) Odds ratios (ORs) with 95% confidence intervals (CIs) for different PRS quantiles, adjusted for age, sex, and asbestos‐exposure. The X‐axis indicates quantiles (≥80th, ≥90th, and >95th), and the Y‐axis represents the ORs. Each point reflects the OR for individuals above the respective threshold.

The internal PRS, constructed with 1123 variants with *p* < 0.001 in the training set, exhibited distinct distributions between PM cases and non‐cancer controls (K–S *p* = 0.0007) (Figure 1A), and disease risk (adjusted for age, sex, and asbestos exposure) increased progressively with higher PRS percentiles. Compared to individuals below each threshold, those in the ≥80th percentile had an OR of 3.46 (95% CI: 1.91–6.53), ≥90th percentile an OR of 4.64 (95% CI: 2.04–11.84), and >95th percentile an OR of 9.54 (95% CI: 2.51–63.11) (Figure 1B). Adding asbestos exposure to a base model with age and sex increased the AUC from 0.61 (95% CI: 0.55–0.67) to 0.71 (95% CI: 0.66–0.77) (DeLong *p* = 0.0002) and including PRS further improved the AUC to 0.75 (95% CI: 0.70–0.80) (DeLong *p* = 0.04).

In the EPIC‐Meso cohort, the PRS did not significantly distinguish incident PM cases from matched controls (K–S *p* = 0.79; *p* = 0.69 after ancestry adjustment), with AUC 0.52 (95% CI: 0.45–0.59) vs. 0.51 (95% CI: 0.44–0.58) for the base model. Although ORs increased at higher percentiles (OR = 1.52, 95% CI: 0.72–3.24 for ≥80th, OR = 1.64, 95% CI: 0.61–4.68 for ≥90th, OR = 2.11, 95% CI: 0.53–10.31 for >95th), none were statistically significant.

To assess predictive validity in a large, phenotyped cohort, we evaluated the internal‐PRS in the UKBB. Among individuals with PM (ICD‐10:C45.0; *n* = 449, 430,984 controls), PRS distribution differed significantly from controls (K–S *p* = 0.02). Risk increased with higher PRS percentiles, with an OR = 1.18, 95% CI: 0.94–1.47, *p* = 0.15 for ≥80th, OR = 1.28, 95% CI: 0.96–1.69, *p* = 0.08 for ≥90th, and OR = 1.56, 95% CI: 1.08–2.17, *p* = 0.01 for ≥95th percentile. When including all mesothelioma cases (ICD‐10:C45; *n* = 547, 430,886 controls), the distribution remained significantly different between cases and controls (K–S *p* = 0.0099). The odds ratio was 1.17 (95% CI: 0.96–1.43, *p* = 0.12) for ≥80th, increasing to 1.34 (95% CI: 1.04–1.71, *p* = 0.02) for ≥90th, and 1.62 (95% CI: 1.17–2.19, *p* = 0.002) for ≥95th percentile. These findings suggest a consistent, though modest, PRS signal across cohorts.

Meta‐PRS, derived from the UKBB‐FinnGen meta‐GWAS with 93 variants (*p* < 1 × 10^−4^), showed consistent directionality and significant discrimination in the full retrospective case–control study (K–S *p* = 0.0002). Incorporating this PRS into a model with age, sex, and asbestos‐exposure modestly improved AUC from 0.72 (95% CI: 0.69–0.76) to 0.74 (95% CI: 0.71–0.78) (DeLong *p* = 0.016). Odds‐ratios rose from 2.5 (95% CI: 1.64–3.79, *p* = 1.99 × 10^−5^) at the 80th percentile to 12.3 (95% CI: 5.67–31.22, *p* = 5.23 × 10^−9^) at the 90th percentile. In EPIC‐Meso, the meta‐PRS showed a similar risk gradient although it did not reach statistical significance (K–S *p* = 0.5).

**FIGURE 2:**
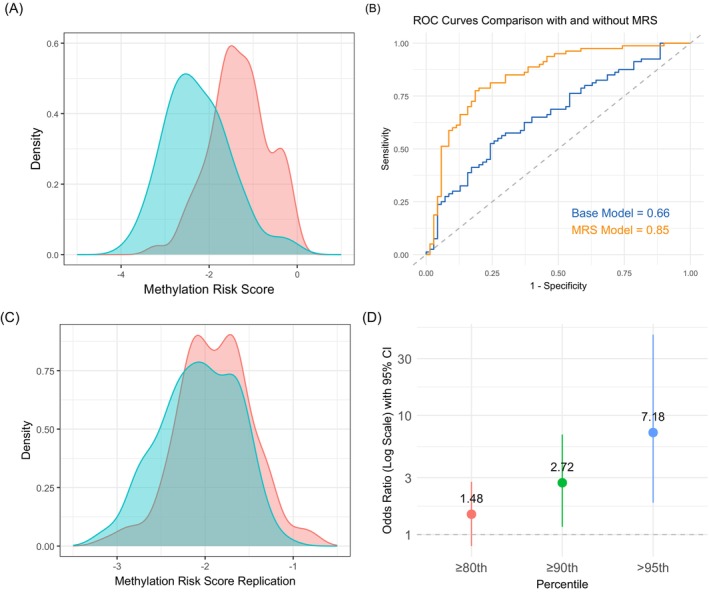
Methylation risk score (MRS) performance and replication across cohorts. (A) Density plot showing MRS distribution for cases (red) and controls (blue) in the retrospective case–control study. The group difference was significant (K–S test *p* = 4.17 × 10^−12^). (B) ROC curves comparing models with and without MRS in the retrospective test‐set. The base model (blue) includes age, sex, and asbestos‐exposure; the MRS model (orange) adds MRS as a predictor. (C) Density plot of MRS for cases and controls in the EPIC‐Meso cohort. The MRS signal was replicated (K‐S test *p* = 0.008). (D) Odds ratios (ORs) with 95% CIs for mesothelioma risk across MRS quantiles (≥80th, ≥90th, and >95th) in the EPIC‐Meso cohort, adjusted for age, sex, and asbestos‐exposure. The Y‐axis is shown on a logarithmic scale and represents the ORs with each point reflecting the OR for individuals above the respective threshold.

The MRS, constructed in the retrospective test‐set using 68 differentially methylated CpGs identified in the training set, outperformed the PRS in both accuracy and significance. It showed stronger distinction between cases and controls (K–S *p* = 4.17e‐12; Figure 2A) and increased AUC from 0.66 (95% CI: 0.58–0.75) to 0.85 (95% CI: 0.78–0.91) when added to a base model including age, sex, and asbestos exposure (DeLong *p* = 3.28 × 10^−5^; Figure 2B). Risk was significantly higher (OR = 10, 95% CI 3.21–44.30, *p* = 3.78 × 10^−4^) for individuals in the ≥80th percentile. While risk appeared to rise at higher MRS percentiles, small sample sizes, particularly among controls, limited the reliability of estimates (e.g., at the 90th percentile, *n* = 13 cases, 2 controls).

Replication of the MRS in the EPIC‐Meso cohort confirmed a significantly different distribution between cases and controls (K–S *p* = 0.008; Figure 2C). Including MRS in a model with age, sex, and asbestos exposure increased the AUC from 0.51 (95% CI: 0.44–0.58) to 0.62 (95% CI: 0.55–0.69) (DeLong *p* = 0.02). The OR increased from 1.48 (95% CI 0.80–2.77, *p* = 0.21) at the 80th percentile to 2.72 (95% CI 1.16–6.91, *p* = 0.03) at the 90th percentile and 7.18 (95% CI 1.85–47.64, *p* = 0.01) at the 95th percentile (Figure 2D). Although we noted a slight upward trend in MRS values among individuals closer to the diagnosis, this difference was not statistically significant (Figure S7).

**FIGURE 3:**
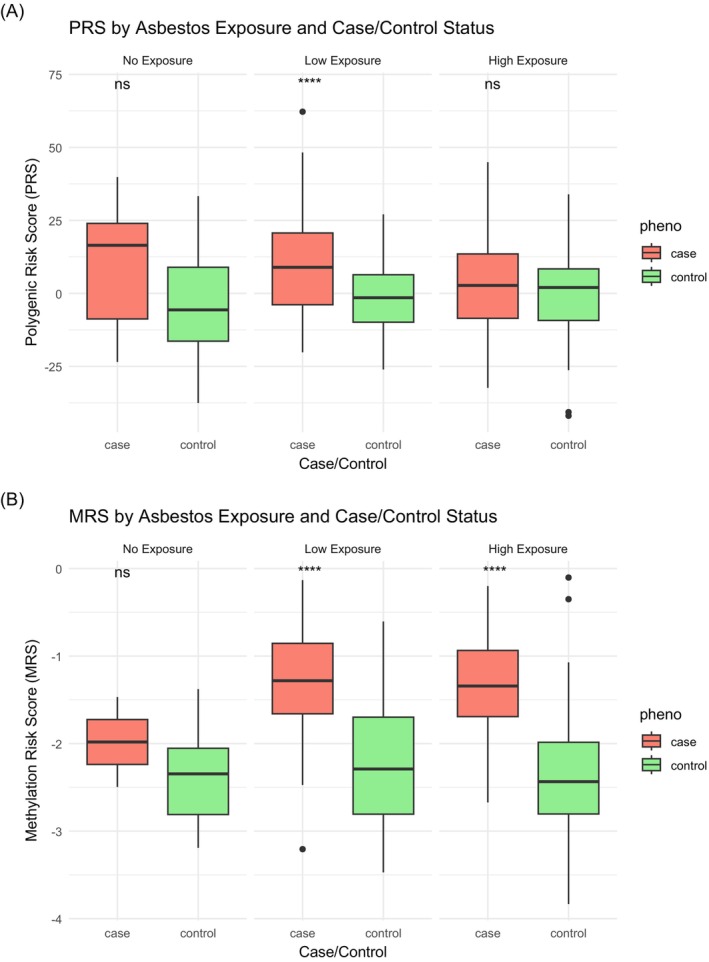
PRS and MRS distributions by exposure and PM status. Box plots showing the distribution of (A) polygenic risk score (PRS) and (B) methylation risk score (MRS) across asbestos‐exposure levels and case/control status in the retrospective case–control study. Asbestos‐exposure is categorized into three levels: 0 (no exposure), 1 (low), and 2 (high). Separate boxplots are shown for cases (red) and controls (green) within each exposure category. Statistical significance was assessed using the Wilcoxon rank‐sum test: ****p < 0.0001; **p < 0.01; ns = not significant (*p* ≥ 0.05).

We then compared the PRS and MRS distributions between cases and controls within each exposure group using Wilcoxon tests. In the retrospective test‐set, PRS values were higher in cases; however, the difference was significant only in the group with low‐exposure (*p* < 0.0001; Figure 3A). In the group with no observed exposure, although the mean PRS was higher in cases, the confidence intervals were wide and overlapping due to small number of cases (*n* = 11). In the high‐exposure group, case and control means were similar (Table S10A). Predictive performance was also highest in the low‐exposure group (AUC = 0.76, 95% CI: 0.69–0.84), compared to the high‐exposure group (AUC = 0.61, 95% CI: 0.51–0.70; DeLong *p* = 0.01), suggesting that PRS may be more informative when exposure is low.

MRS values in the retrospective test‐set were significantly higher in cases for both low‐ and high‐exposure groups (*p* < 0.0001). In the no‐exposure group, the mean MRS was higher in cases but numbers were limited (*n* = 2) and the difference was not statistically significant (Figure 3B; Table S10B). Stratified logistic regression confirmed that MRS significantly increased disease risk in the group with low‐exposure (OR = 8.76, 95% CI: 3.63–27.01, *p* < 0.001) and with high‐exposure (OR = 6.08, 95% CI: 2.37–20.18, *p* < 0.001), possibly also reflecting some methylation changes linked to disease presence, with an AUC of 0.84 in both low (95% CI: 0.7618–0.9265) and high (95% CI: 0.7141–0.9693). Overall evidence from the retrospective case–control study suggests that at high asbestos‐exposure levels, environmental risk may dominate over genetic contribution.

In EPIC‐Meso, PRS did not reach significance in any of the exposure groups (Figure S8A, B; Table S11A). MRS showed consistent trends across exposure strata, with higher scores in cases than in controls and maintained significant associations in both the no‐ and low‐exposure groups (*p* = 0.01). In the group with high exposure, the mean difference was smaller (−1.98 vs. –2.14) (Table S11B) and the confidence intervals overlapped (*p* = 0.16), although the direction of effect remained consistent. A formal test of interaction between MRS and asbestos exposure level showed no significant interaction (*p* = 0.98 for low, *p* = 0.31 for high), indicating that the varying levels of significance across strata are likely due to differences in sample size or effect magnitude rather than a true modifying effect of exposure.

In subgroup analyses, the Meta‐PRS showed consistent trends across exposure strata in the full retrospective dataset (Figure S9A). PRS values were higher in cases than controls within the low‐exposure group (*p* = 0.003) and showed a similar, though non‐significant, trend in the no‐exposure group (*p* = 0.096; 17 cases, 107 controls) (Table S12A). Predictive performance was highest in the no‐exposure (AUC = 0.72, 95% CI: 0.58–0.86) and low‐exposure groups (AUC = 0.69, 95% CI: 0.63–0.74), and lower in the high‐exposure group (AUC = 0.64, 95% CI: 0.57–0.70). Although effect directions were consistent, the differences between groups were not statistically significant.

The reduced performance at higher exposure levels likely reflects differences in the base GWAS design. The UKBB‐FinnGen meta‐analysis did not adjust for asbestos‐exposure, averaging effects across populations with unknown exposure distributions, whereas the internal exposure‐adjusted GWAS captured context‐specific genetic effects, more evident in lower exposure groups. A similar pattern was observed in the EPIC‐Meso cohort, with cases showing higher Meta‐PRS values than controls in the low‐exposure group (*p* = 0.04) and a comparable but non‐significant trend in the no‐exposure group (*p* = 0.07), while no difference was observed at high‐exposure (*p* = 0.16) (Figure S9B; Table S12B).

In a subset of 64 individuals from the retrospective test‐set with both genotype and methylation data (35 cases and 29 controls), we built four logistic regression models: PRS only, MRS only, a combined PRS + MRS model, and a model including the PRS × MRS interaction term. Addition of the PRS to a baseline model including age and sex increased the AUC from 0.65 (95% CI: 0.51–0.78) to 0.73 (95% CI: 0.61–0.85) after accounting for exposure, indicating a modest improvement in discrimination (DeLong *p* = 0.21). Adding MRS significantly increased the AUC to 0.91 (95% CI: 0.83–0.98; DeLong *p* = 0.005). The interaction model did not further enhance the AUC beyond the combined model; however, pseudo R^2^ values suggested that the interaction may explain additional variability (Figure S10). Using a threshold at the 80th percentile of PRS, we observed 80% specificity and 20% sensitivity. Incorporating MRS improved the specificity and sensitivity to 93% and 31%, respectively, in this subset.

Extending the integrated risk score analysis to the EPIC‐Meso cohort (*n* = 264) yielded similar results. The PRS model showed no improvement over the base model with AUC = 0.52 (95% CI: 0.45–0.59) versus 0.51 (95% CI: 0.44–0.58). In contrast, the MRS model significantly improved the performance (AUC = 0.61 [95% CI: 0.54–0.68]; DeLong *p* = 0.03). The combined PRS + MRS model and the interaction model (PRS × MRS) showed similar AUCs of 0.61 (95% CI: 0.54–0.68) and 0.62 (95% CI: 0.55–0.69), respectively, both significantly better than the base model (*p* < 0.05), but not significantly different from the MRS‐only model (*p* = 0.83 and *p* = 0.56, respectively). Pseudo R^2^ values indicated that the interaction model explained slightly more of the variability (Figure S11). Adding MRS to the EPIC‐Meso cohort showed consistent improvements with specificity increasing from 82% to 85% and sensitivity from 22% to 24% among individuals above the 80th percentile for MRS. Notably, 56% of individuals above this threshold were PM cases, rising to 70% in the ≥90th percentile group.

To explore the effect of risk levels separately within each asbestos‐exposure group in the combined model, we categorized individuals into low‐, intermediate‐, and high‐risk groups based on the 33rd and 67th percentiles of both PRS and MRS. Logistic regression was performed within each exposure group, using the intermediate group as the reference. In the low‐exposure group, high‐risk individuals had significantly increased odds of PM (OR = 3.98, 95% CI: 1.13–18.68, *p* = 0.045). No significant associations were observed in the no‐exposure (OR = 4.27, 95% CI: 0.55–88.63, *p* = 0.22) or high‐exposure groups (OR = 0.46, 95% CI: 0.06–2.51, *p* = 0.39). The AUC was higher in the low‐exposure group (AUC = 0.61, 95% CI: 0.50–0.75) than in the high‐exposure group (AUC = 0.54, 95% CI: 0.46–0.62), although this difference was not statistically significant (DeLong *p* = 0.20).

A net reclassification index (NRI) analysis, categorizing predicted probabilities into low (<20%), intermediate (20%–80%), and high (>80%) risks, demonstrated that the combined PRS‐MRS model improved classification accuracy, with an overall NRI of 0.2273, *p* = 0.001 (cases: 0.1128; controls: 0.1145). The overall NRI of approximately 23% demonstrated a significant enhancement in predictive accuracy, suggesting that the combined PRS + MRS model more effectively stratified both high‐risk and low‐risk individuals compared to the base model with only age, sex, and asbestos exposure.

### 3.5 SNP‐exposure interaction analysis

SNP‐exposure interaction analyses identified variants with potential differential effects based on asbestos exposure. Full results, including detailed SNP associations, stratified analyses, and RERI calculations, are provided in Data S1, Tables S13–S15.

## 4 DISCUSSION

This study explored the genetic and epigenetic contributions to PM risk by focusing on the interplay between PRS, MRS, and asbestos exposure. The findings support the complexity of PM as a disease influenced by both environmental and inherited factors.^12^, ^13^, ^32^, ^33^

The GWAS identified variants associated with PM risk and broader cancer biology. Asbestos exposure was included in the GWAS model to reduce residual variance, although it was not a classical confounder. Notably, variants in *OAT*, *ITGBL1*, *PLA2R1*, *MICAL3*, *PPP2R2B*, *TPK1*, and *PCDH7* may contribute to PM pathogenesis. *OAT* and *ITGBL1* are overexpressed in mesothelioma and other cancers, promoting oncogenic metabolism and tumor aggressiveness.^34^, ^35^, ^36^, ^37^, ^38^ *PLA2R1* functions as a tumor suppressor,^39^ while *MICAL3* regulates cytoskeletal remodeling and is linked to poorer clinical outcomes.^40^ *PPP2R2B* and *PCDH7* are associated with chemotherapy resistance and lung cancer risk,^41^, ^42^, ^43^, ^44^, ^45^ and *TPK1*, essential for thiamine metabolism, is upregulated in cancers potentially meeting the metabolic demands observed in PM.^46^, ^47^ Functional enrichment highlighted pathways in amino acid and thiamine metabolism, cell adhesion, cytoskeletal organization, and immune regulation, processes relevant to early tumor development. GRASP analyses linked several variants to other cancers (including colorectal, breast, and prostate cancers), indicating broader relevance in cancer susceptibility.

SNP‐exposure interaction analysis revealed complex patterns. While direct SNP‐asbestos interaction terms were not statistically significant, stratified analyses showed that some variants (e.g., rs12900352, rs2440012) increased the risk in exposed individuals, whereas others (e.g., rs2122342) were less represented in cases compared to controls across exposure groups. RERI analyses indicated antagonistic interactions between exposure and several SNPs, suggesting that certain genetic factors may mitigate the carcinogenic effects of asbestos. These findings warrant further investigation with larger cohorts.

The internal PRS performed well in the retrospective case–control study, particularly among individuals with low asbestos‐exposure, suggesting that genetic susceptibility may be more detectable when environmental exposure is limited, a finding that is consistent with a previous study.^48^ The PRS also showed a modest but consistent polygenic signal in the UKBB where odds ratios increased progressively across higher PRS percentiles in both pleural and all mesothelioma definitions. These findings support the relevance of the PRS, even though its immediate clinical utility remains limited without additional context or stratification linked to asbestos‐exposure.

To strengthen the evidence, we built a Meta‐PRS from the UKBB–FinnGen meta‐GWAS summary statistics and tested it in the full retrospective case–control dataset. The Meta‐PRS was significantly associated with PM risk (K–S *p* = 0.0002), supporting the reproducibility of the polygenic component identified internally. The consistency across datasets suggests that some common genetic background for PM can be detected, even though the total predictive power remains modest.

Validation in EPIC‐Meso showed a similar risk gradient but did not reach statistical significance likely due to heterogeneity in exposure history, ancestry, and study design. EPIC includes highly exposed cases with diverse types of asbestos exposures and more heterogeneous controls. In and its smaller, nested case–control structure reduces power, factors that may mask the genetic signal observed in the more homogeneous retrospective dataset.

The MRS outperformed the PRS, significantly distinguishing cases from controls and improving AUC from 0.66 to 0.85 in the test set. It replicated well in the EPIC‐Meso cohort (AUC: 0.51 to 0.62) and maintained strong associations with PM risk across exposure levels, with no significant interaction with asbestos exposure. These findings indicate that MRS captures early epigenetic changes, likely independent of asbestos exposure dose, and may serve as a broadly applicable biomarker across populations.

Integrating PRS and MRS enhances risk stratification. In the retrospective subset of 64 individuals with both data types, the combined model achieved an AUC of 0.91. Compared to PRS alone, incorporating MRS increased both specificity and sensitivity (80% to 93% and 20% to 31%, respectively). While external validation in the EPIC‐Meso cohort showed modest gains (AUC ~ 0.61), the dual approach still improved specificity (82% to 85%) and sensitivity (22% to 24%), and consistently improved overall risk reclassification, as reflected by the NRI of ~23%.

Collectively, these findings underscore the value of combining genetic and epigenetic data when modeling PM risk, a disease that is strongly influenced by asbestos exposure. While PRS captures inherited patterns, its limited portability highlights the challenges of population‐specific genetic models.^49^, ^50^ In contrast, the robustness of MRS across exposure strata and replication in the prospective EPIC‐Meso cohort suggests that epigenetic alterations offer a more stable indicator of early biological changes, driven by both genetic and environmental factors.

Clinically, MRS may serve as a more effective first‐line screening tool in exposed individuals, whereas PRS can be used to further refine risk estimates. This dual strategy leverages the strengths of both measures to enhance overall risk stratification and, combined with other biomarkers (e.g., mesothelin, calretinin, and PM‐related miRNAs), could inform targeted monitoring programs and early intervention strategies, particularly among individuals with known asbestos exposure.

Despite promising results, this study has some limitations. The relatively small sample sizes and the challenges inherent in studying rare diseases may limit the generalizability of our results. The rarity and long latency of PM also reduce the positive predictive value in unselected populations, suggesting that risk models may be most informative in asbestos‐exposed subgroups to obtain meaningful absolute risk increases. Moreover, PRS typically show reduced predictive power across ancestries, requiring recalibration in larger and more diverse cohorts, whereas MRS appears more transferable. Cross‐dataset validation can help mitigate overfitting, yet only one prior mesothelioma GWAS with publicly available summary statistics exists.^12^, ^51^ Broader replication remains limited by the lack of large genotyped case collections. To address this, efforts are underway to combine available datasets with UKBB and FinnGen to increase power and refine variant‐level associations, which can enable deeper investigation of shared genetic architecture in future work.

In conclusion, our findings demonstrate that while genetic factors may contribute to PM risk alongside asbestos exposure, the dynamic nature of epigenetic alterations, likely triggered by asbestos and the activation of carcinogenic processes, offers a robust complementary marker. Given the lack of standalone biomarkers for early detection, the integration of PRS and MRS holds promise for enhancing risk prediction and paves the way for combining these with other biomarkers and detailed asbestos exposure data for a more comprehensive and personalized screening approach that could ultimately improve early intervention and clinical outcomes.

## AUTHOR CONTRIBUTIONS

**Khadija Sana Hafeez:** Conceptualization; methodology; software; data curation; formal analysis; visualization; validation; writing – original draft; project administration; resources. **Carla Debernardi:** Validation; writing – review and editing; data curation. **Alessandra Allione:** Data curation; writing – review and editing. **Elton Jalis Herman:** Data curation; writing – review and editing. **Simonetta Guarrera:** Data curation; writing – review and editing. **Daniela Ferrante:** Data curation; writing – review and editing. **Anna Aspesi:** Data curation; writing – review and editing. **Marika Sculco:** Data curation; writing – review and editing. **Marta La Vecchia:** Data curation; writing – review and editing. **Carlotta Sacerdote:** Data curation; resources; writing – review and editing. **Federica Grosso:** Writing – review and editing; data curation; resources. **Christina M. Lill:** Data curation; resources; writing – review and editing. **Giovanna Masala:** Data curation; resources; writing – review and editing. **Marcela Guevara:** Data curation; resources; writing – review and editing. **Matthias B. Schulze:** Data curation; resources; writing – review and editing. **Salvatore Panico:** Data curation; resources; writing – review and editing. **Yaszan Asgari:** Writing – review and editing; data curation; resources. **Seehyun Park:** Data curation; resources; writing – review and editing. **Giovanna Tagliabue:** Writing – review and editing; data curation; resources. **Anne Tjønneland:** Writing – review and editing; data curation; resources. **Antonio Agudo:** Writing – review and editing; data curation; resources. **Elisabete Weiderpass:** Writing – review and editing; data curation; resources. **Corrado Magnani:** Writing – review and editing; data curation; resources. **Irma Dianzani:** Data curation; resources; writing – review and editing. **Paolo Vineis:** Data curation; resources; writing – review and editing; conceptualization. **Elisabetta Casalone:** Writing – review and editing; data curation; supervision; project administration. **Giuseppe Matullo:** Conceptualization; supervision; project administration; writing – review and editing; funding acquisition.

## CONFLICT OF INTEREST STATEMENT

The authors declare no competing financial interests. Corrado Magnani and Irma Dianzani were expert witnesses for the public prosecutor in court trials on asbestos exposure and related health consequences. F.G. declares, outside the submitted work, to have received travel support and advisory role compensation from BMS, MSD, Novocure, AstraZeneca, Novartis, PharmaMar, and Deciphera. All other authors declare no competing interests.

## ETHICS STATEMENT

The retrospective study was approved by the Ethics Committee of the Italian Institute for Genomic Medicine (30/10/2015; protocol N°CE‐2015‐GM‐2). The study protocol was approved by the Ethics Committee of the Italian Institute for Genomic Medicine. The EPIC‐Meso study protocol was approved by the International Agency for Research on Cancer ethics committee (Lyon, France, IEC 24‐08). Ethics approval for the UK Biobank study was obtained from the North West Centre for Research Ethics Committee (11/NW/0382). UK Biobank data used in this study were obtained under approved application 78537. The present study adhered to the Declaration of Helsinki. All participants provided written informed consent and completed standardized questionnaires.

## Supporting information


**Data S1.** Supporting information.

## Data Availability

The GWAS summary statistics are available through the NHGRI‐EBI GWAS Catalog under study accession number GCST001978. The array data has been deposited in the European Genome‐phenome Archive (EGA), hosted by the EBI and the CRG, under the accession number EGAS00001006432. Additional details about the EGA can be found at https://ega-archive.org and in the publication titled “The European Genome‐phenome Archive of human data consented for biomedical research” available at Nature Genetics. UK Biobank data are available through the procedure described in http://www.ukbiobank.ac.uk/using-the-resource. FinnGen data are available upon application to (https://www.finngen.fi/en). Further data that support the findings of this study are available after approval by the Data Access Committee, from the corresponding author upon request.
