# Supplementary material for: Integrating polygenic and methylation risk scores for pleural mesothelioma risk stratification
Source: Int J Cancer. 2025 Dec 30;158(11):2866–79. doi: 10.1002/ijc.70316 (PMC13047241; doi:10.1002/ijc.70316)
Supplement: Supplementary file 1 — Data S1. Supporting information. [file IJC-158-2866-s001.pdf]

Supplementary Material for Manuscript: Integrating Polygenic and Methylation Risk Scores for Pleural Mesothelioma risk stratification

Khadija Sana Hafeez, Carla Debernardi, Alessandra Allione, Elton Jalis Herman, Simonetta Guarrera, Daniela Ferrante, Anna Aspesi, Marika Sculco, Marta La-Vecchia, Carlotta Sacerdote, Federica Grosso, Christina M. Lill, Giovanna Masala, Marcela Guevara, Matthias B. Schulze, Salvatore Panico, Yaszan Asgari, Seehyun Park, Giovanna Tagliabue, Anne Tjønneland, Antonio Agudo, Elisabete Weiderpass, Corrado Magnani, Irma Dianzani, Paolo Vineis , Elisabetta Casalone, Giuseppe Matullo

Contents

Supplementary Methods: 2

Italian retrospective Cohort and Data Processing 2

EPIC-Meso Cohort and Data Processing..3

UK Biobank Genotyping and Quality Control 4

Demographic Comparison and Statistical Results 5

SNP-Exposure Interaction Analysis..6

SNP-Exposure Interaction Analysis Results 6

Supplementary Figures: 7

Supplementary Tables 17

Supplementary Methods:

Study Cohorts

Italian retrospective Cohort and Data Processing

The Italian study population is part of a broader, collaborative effort investigating malignant pleural mesothelioma (MPM) in high-risk regions of Northern Italy. Subjects were recruited from registry-based case-control studies conducted in Casale Monferrato, Turin, Genoa, and La Spezia, areas historically impacted by occupational and environmental asbestos exposure.

Casale Monferrato Panel
This population-based study recruited histologically confirmed MPM cases diagnosed between 2001 and 2010, residing in the local health authority area. Casale Monferrato experienced decades of widespread asbestos exposure due to the presence of a cement-asbestos factory active from 1907 to 1986. Controls were randomly selected from local health registries and matched 2:1 to cases by age (±18 months) and sex.

Turin Panel
The hospital-based Turin study enrolled MPM cases admitted between 2004 and 2006 to major hospitals in the area. Controls were selected from general medicine or urology patients without neoplastic or respiratory conditions, matched 1:1 to cases by age (±24 months) and sex. All participants resided in the Turin area at the time of enrolment.

Liguria Panel (Genoa and La Spezia)
MPM cases were recruited between 1999 and 2006 through the Cancer of RESpiratory Tract (CREST) biobank. Controls included healthy volunteers or hospital patients with non-neoplastic, non-respiratory conditions, matched to cases by age and sex. Liguria has high MPM incidence due to historical shipyard and asbestos-intensive industries.

Asbestos Exposure Assessment
Asbestos exposure was evaluated by an expert industrial hygienist (D.M.) based on detailed work histories, and categorized as “no/unlikely,” “low,” or “high.” More granular exposure classifications (e.g., certain/probable occupational, household, environmental) were also collected.

Across all panels, blood samples were collected prior to treatment and processed for DNA extraction. Participants provided informed consent and completed standardized questionnaires covering demographics, occupational history, and lifestyle.

DNA Extraction and Genotyping
Genomic DNA was extracted from 200 µL of whole blood using the QIAamp DNA Blood Mini Kit (QIAGEN). DNA quality was assessed via agarose gel electrophoresis and NanoDrop spectrophotometry. For genotyping, 200 ng of DNA per sample was used on Illumina BeadChip arrays. A total of 720 samples were genotyped using the HumanCNV370-Quad BeadChip, and an additional 80 samples were analyzed using the Human610-Quad BeadChip, which includes full coverage of the CNV370 panel. Genotype calling was performed using GenomeStudio V2011.1 with Genotyping Module v1.9.4 (Illumina Inc.).

Following quality control, 749 individuals (387 cases and 362 controls) with high-quality genotype and phenotype data were available for the analysis.

As a part of these steps, Individuals with more than 5% of genotype missingness were removed [n=19 for 740 cohort and 0 for 80 cohort]. SNPs with a missing call rate of more than 5% across all individuals were also removed [n= 5370 for 740 cohort and 4903 for 80 cohort]. The dataset was limited to SNPs that were in Hardy-Weinberg equilibrium (HWE), where SNPs deviating from HWE at p<1e-6 in controls and at p<1e- 10 in cases were removed [n=9371 for 740 cohort and 8870 for 80 cohort]. Since PM is a rare disease, markers with a Minor Allele Frequency (MAF) < 0.5% [n= 2574 for 740 cohort and 0 for 80 cohort] were omitted. This threshold is selected in order to retain variants that are rare enough to potentially have functional effects, but still provide enough coverage for statistical analysis for rare diseases like PM. Related individuals in the overall samples (up to third degree) were inferred from their genotypes (those with an IBD above 0.125) and removed [n=23]. Samples who deviate more than 3 standard deviations from the heterozygosity rate mean were also removed [n=8]. Moreover 8 samples with lacking exposure/clinical information were also excluded.

DNA Methylation Subset and Processing
A subset of subjects from the Italian genotyped cohort was selected for DNA methylation profiling, drawn from ongoing studies in Casale Monferrato and Turin. Participants were histologically confirmed PM cases and matched controls recruited between 2000 and 2010. Controls were either population-based (Casale Monferrato) or hospital-based non-neoplastic, non-respiratory patients (Turin). Inclusion in the methylation study required: (i) availability of high-quality DNA, (ii) documented asbestos exposure level, and (iii) exposure above background, as defined previously.

Genomic DNA was extracted from peripheral whole blood using the QIAamp DNA Blood Mini Kit (QIAGEN). DNA quantity and purity were assessed by NanoDrop 8000 Spectrophotometry, and integrity confirmed via agarose gel electrophoresis. For methylation profiling, 500 ng of DNA per sample was bisulfite converted (EZ-96 DNA Methylation-Gold Kit, Zymo Research). Genome-wide DNA methylation was assessed using the Infinium HumanMethylation450 BeadChip (Illumina), interrogating >485,000 CpG sites.

Cases and controls were randomly distributed across plates and BeadChips. Samples were also randomized by position to minimize and test for potential position effects; no position bias was observed. BeadChips were processed per manufacturer’s protocol. Signal detection and quality control were performed in GenomeStudio v2011.1 with the Methylation Module v1.9.0, using over 200 internal control probes to assess assay performance.

Data preprocessing was conducted using the R package methylumi. Beta values were calculated as the ratio of methylated signal to total signal at each CpG site. Quality filters excluded: (i) CpG sites with detection p-value ≥ 0.01; (ii) probes missing in >20% of samples; (iii) probes overlapping SNPs with MAF ≥ 0.05 (CEU); (iv) samples with call rate ≤ 95%; and (v) all CpGs on sex chromosomes. An additional 50,186 CpG probes were removed based on Zhou et al.

EPIC-Meso Cohort and Data Processing

The European Prospective Investigation into Cancer and Nutrition (EPIC) is a large, multicentre cohort study conducted across 23 centres in 10 European countries, designed to investigate the role of

biological, lifestyle, and environmental factors in cancer and chronic disease development. Between 1993 and 1998, EPIC enrolled 521,468 healthy individuals who have since been followed longitudinally.

Within the EPIC cohort, 134 individuals developed malignant pleural mesothelioma (PM), referred to here as the EPIC-Meso cohort. Diagnosis was based on cancer registry or clinical follow-up data and classified using ICD-10 code C38.4 (malignant neoplasm of pleura). Follow-up data were centralized at IARC between 2014 and 2016, with an average time to diagnosis of 8.3 years (range: 0.5-18.8 years). Controls were individually matched 1:1 to cases based on sex, age at enrolment (±1.5 years), study centre, and asbestos exposure classification.

Genotyping was performed using the Infinium Global Screening Array-24 v3.0/OmniExpress platform (Illumina Inc., San Diego, CA). Genotype calling was conducted using GenomeStudio software (Illumina).

DNA methylation (DNAm) levels were measured from buffy coat samples collected at enrolment using the Infinium MethylationEPIC BeadChip (>850,000 CpG sites; Illumina, San Diego, CA). Laboratory methods for DNA extraction, array processing, and quality control were performed according to standardized protocols and previously published methods.

Asbestos Exposure Assessment in EPIC-Meso
Occupational history was obtained from the baseline EPIC questionnaire, including current and past employment. Exposure was assessed using a semi-quantitative job-exposure matrix (JEM), developed by expert epidemiologists as previously described [15], assigning each of 52 job categories a score for exposure probability (0–3) and intensity (0–3). These scores were multiplied to calculate an Exposure Index (EI). For the present study, asbestos exposure was treated as a categorical variable with three levels, No exposure: EI = 0, Low exposure: EI = 1-3, High exposure: EI ≥ 4.

UK Biobank Genotyping and Quality Control

Two arrays were used to genotype UK Biobank participants. The UK BiLEVE Axiom Array was used for 49,950 participants, while the Applied Biosystems UK Biobank Axiom Array was used for the remaining 438,427 participants. Principal Component Analysis (PCA) was performed on the genetic data, and centralized quality control (QC) was conducted on individuals identified as part of the largest genetic cluster (N = 463,844), as determined by Aberrant, an unsupervised clustering algorithm. Variants were assessed for allele frequency variation across technical variables (batch, plate, sex, array) and for deviations from Hardy-Weinberg Equilibrium (HWE), using stringent p-value thresholds ($< 10^{-12}$). Variants that failed any of these criteria within a given batch were set to missing.

Imputation was performed using IMPUTE4 for 487,442 individuals. The reference panel included both the Haplotype Reference Consortium (HRC) and a merged UK10K + 1000 Genomes panel. Only SNPs present in both panels were retained in the final imputed dataset, resulting in a total of 96,959,328 SNPs.

Demographic Comparison and Statistical Results

Gender Distribution (Fisher's Exact Test)
Fisher’s exact test was used to assess the gender distribution between the retrospective and EPIC-Meso cohorts. No statistically significant difference was observed in the distribution of males and females between the two cohorts. Specifically, for males, the p-value was 0.6, and for females, the p-value was 1.0, indicating no significant difference in gender distribution between the cohorts.

Age Comparison (t-test)
To compare the mean age between males and females in the Retrospective cohort and the EPIC-Meso cohort, we performed independent t-tests for each group. In the Retrospective cohort, the mean age of males was significantly higher than in the EPIC-Meso cohort for both the cases and controls (p-value < 0.0001). This significant difference reflects the older age of the retrospective cohort. Similarly, in the Retrospective cohort, the mean age of females was significantly higher than in the EPIC-Meso cohort (p-value < 0.0001). Again, this suggests that individuals in the retrospective cohort were older compared to those in the EPIC cohort.

In both cases, the significant differences in age between the two cohorts highlight the inherent age gap between the Retrospective cohort (which includes diagnosed cases) and the EPIC-Meso cohort (which includes pre-clinical cases).

Asbestos Exposure Comparison (Fisher's Exact Test)
Fisher’s Exact Test was used to compare the distribution of asbestos exposure (No, Low, High, NA) between the Retrospective cohort and the EPIC-Meso cohort. The results showed a significant difference in overall asbestos exposure between the two cohorts ($p < 0.0001$), with a significant difference also observed in the No Exposure and High Exposure groups ($p = 7.54e-06$ and $p = 0.01171$, respectively). However, there was no significant difference in the Low Exposure group ($p = 1$), suggesting that asbestos exposure is more similar between the two cohorts in individuals with low exposure.

Conclusion:
The gender distribution did not differ significantly between the cohorts, suggesting a similar gender composition across both the groups. However, the significant age difference between the cohorts reflects the inherent age gap between the two study designs, with the retrospective cohort being older. The asbestos exposure results suggest that the two cohorts have different exposure patterns, with the retrospective cohort showing higher exposure levels, especially in the high-exposure group, which may be an important factor in disease development.

SNP-Exposure Interaction Analysis:

Interactions between GWAS-significant SNPs from the retrospective cohort and asbestos exposure were evaluated using logistic regression with codominant genotype coding (0 = homozygous major, 1 = heterozygous, 2 = homozygous minor). We also performed stratified analyses for exposed versus unexposed subjects, excluding variants with a single genotype level in any group. The analyses were adjusted for age, sex, and PCs. We evaluated deviations from an additive model by calculating the relative excess risk due to interaction (RERI), defined as [$OR_{11} - OR_{01} - OR_{10} + 1$]. Here, OR_{11} is the odds ratio for individuals carrying the SNP and exposed to asbestos, OR_{10} for exposed non-carriers, and OR_{01} for SNP carriers who were unexposed. Asbestos exposure was dichotomized (exposed vs. unexposed). Under the null hypothesis, RERI ≈ 0; values >0 indicate an additive interaction, whereas a negative RERI suggests an antagonistic (sub-additive) interaction.

SNP-Exposure Interaction Analysis Results:

Interaction analysis between retrospective genome wide significant variants and asbestos exposure are shown in Supplementary Table 13-15.

Three SNPs (rs2459219, rs12822999, rs1351034) were significantly associated with increased PM risk, and rs2122342 showed a significant protective effect (OR = 0.24, 95% CI: 0.10-0.62, p = 0.003). However, the SNP-exposure interaction terms did not reach statistical significance (Supplementary Table 13). Stratified analyses revealed that SNPs such as rs12900352 and rs2440012 were significantly associated with PM risk only in asbestos-exposed individuals ($p < 0.05$), suggesting these variants may predispose individuals to PM under conditions of asbestos exposure (Supplementary Table 14). Other SNPs (rs113946246, rs1469849, rs2490010, rs67206049, rs9539946) exhibited a protective effect in exposed subjects but were not significant in unexposed groups. Three SNPs (rs12822999, rs1351034, rs401224) were significant in both exposure groups and exhibited higher odds ratios in the unexposed group than in the exposed group but with stronger statistical significance in exposed individuals. Notably, rs2122342 maintained a protective association in both groups, although the OR was lower in unexposed individuals. The variant rs1877196 was significant only in the unexposed group. Relative excess risk due to interaction (RERI) analyses (Supplementary Table 15) revealed significant antagonistic interaction effects for six SNPs (rs9539946, rs1469849, rs113946246, rs2490010, rs67206049, rs401224), as indicated by significant negative RERI values. In contrast, the variants rs2122342 and rs1877196 also exhibited negative RERI values, but these did not reach statistical significance.

Supplementary Figures:

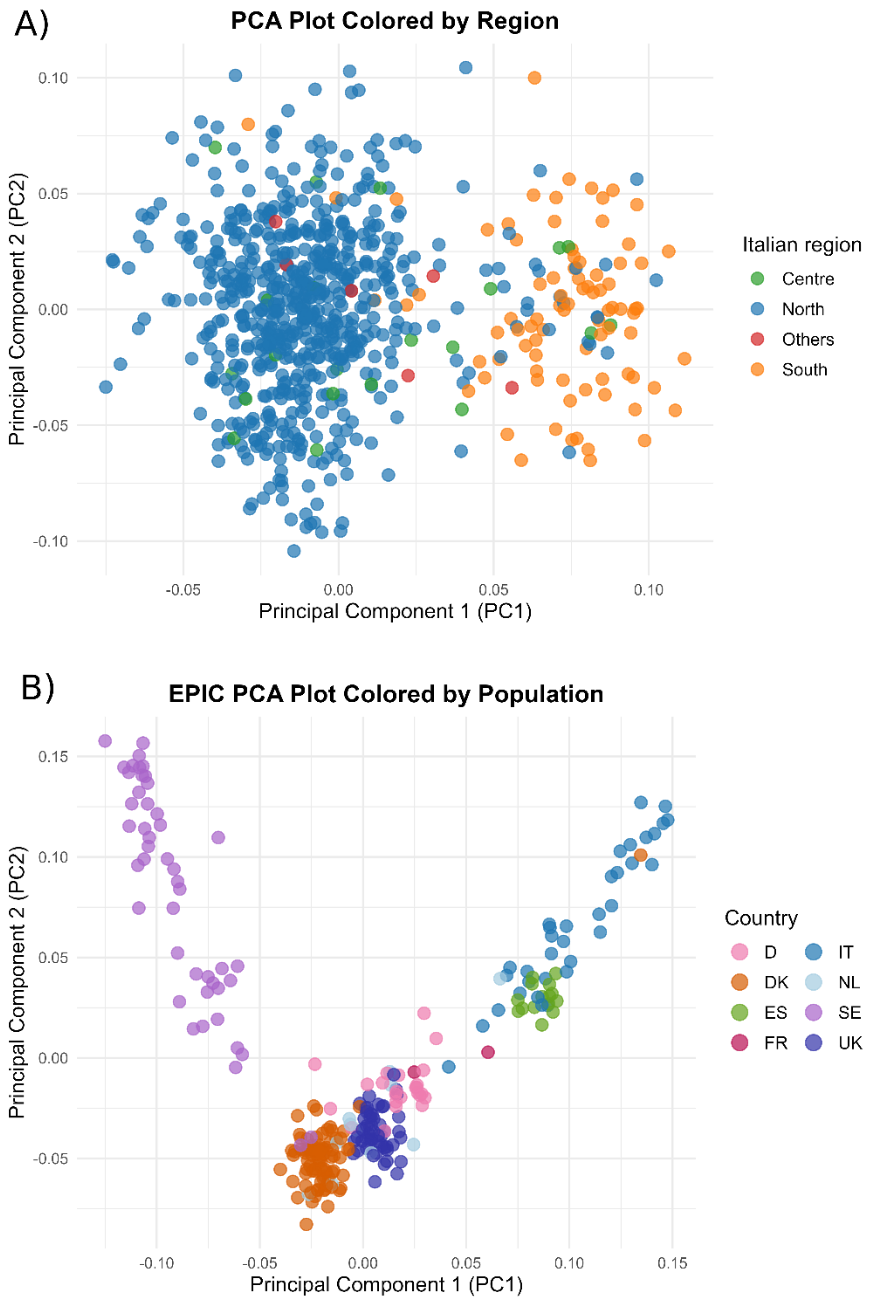

Supplementary Figure S1.
Principal Component Analysis (PCA) of the Retrospective and EPIC-Meso Cohorts.
PCA Plot for the Retrospective Cohort (A) and the EPIC-MESO cohort (B). The x-axis (PC1) and y-axis (PC2) represent the top two principal components capturing genetic variation, and each point corresponds to an individual. Colors indicate different population regions, highlighting the underlying population structure.

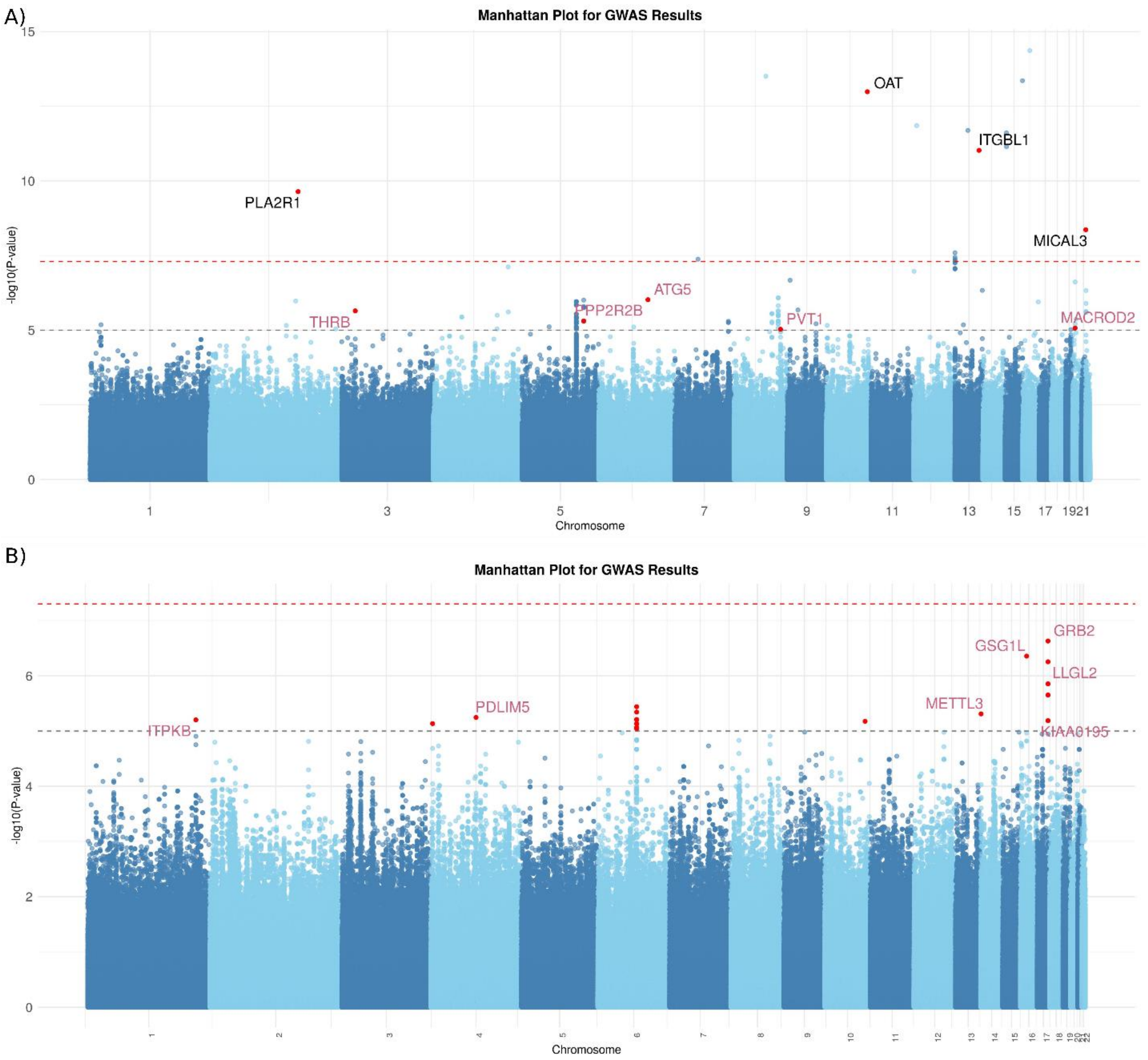

Supplementary Figure S2.
Manhattan Plots of GWAS Results for Retrospective and EPIC-Meso Cohorts.
Manhattan Plot for the A) Retrospective and B) EPIC-Meso cohort with annotated protein-coding genes. Each point represents a single SNP, plotted by genomic position (x-axis) and $-\log_{10}$(p-value) (y-axis). The red line indicates the genome-wide significance threshold ($p = 5 \times 10^{-8}$); the blue line represents suggestive significance ($p = 1 \times 10^{-5}$).

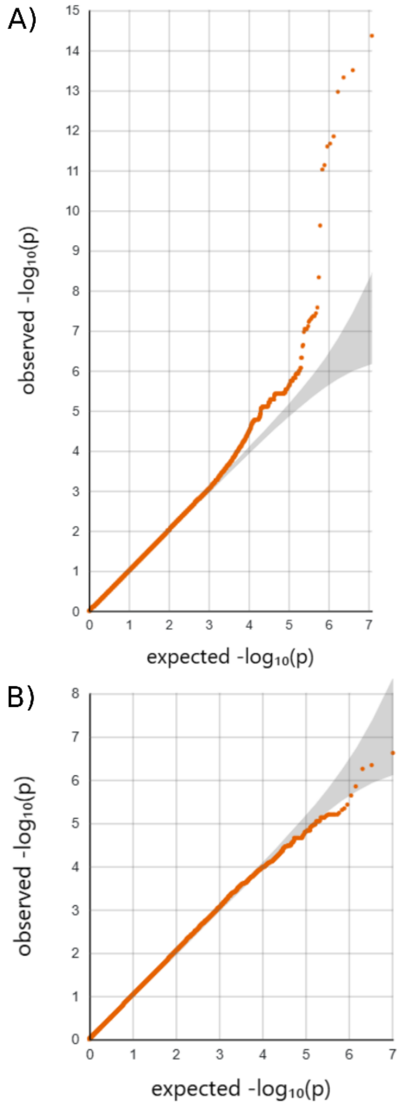

Supplementary Figure S3.
Quantile-Quantile (QQ) Plots of GWAS p-values in Retrospective and EPIC-Meso Cohorts.
Observed vs. expected -$\log_{10}$(p-values) are shown to evaluate inflation and deviation from the null distribution for the (A) Retrospective Cohort and the (B) EPIC-MESO cohort with lambda genomic inflation factors, GC λ_1 = 1.018 and GC λ_2 =1.059 respectively.

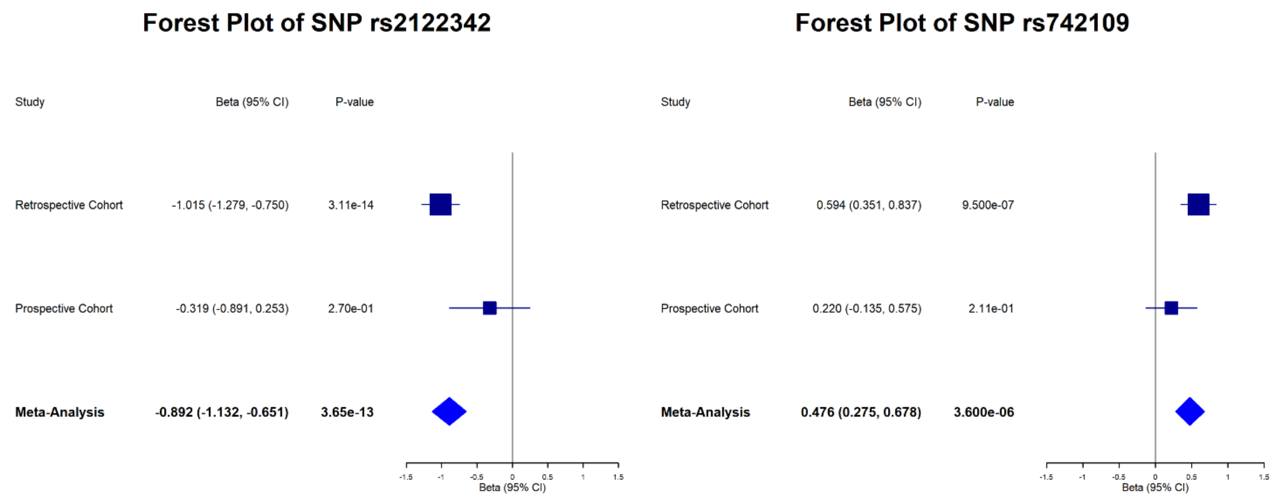

Supplementary Figure S4.
Forest Plots of SNP Associations for rs2122342 and rs742109 in Retrospective, EPIC-Meso, and Meta-analysis Results.
Forest plot for SNP rs2122342 and rs742109 showing the association with PM risk in retrospective and EPIC-Meso cohorts, and the meta-analysis summary. The retrospective and EPIC-Meso cohort results are shown with effect sizes (Beta), 95% confidence intervals (CIs), and p-values, followed by the combined meta-analysis results. Squares represent cohort-specific effect sizes, with the size proportional to the study weight, while diamonds represent the pooled effect from meta-analysis. Horizontal lines indicate 95% CIs.

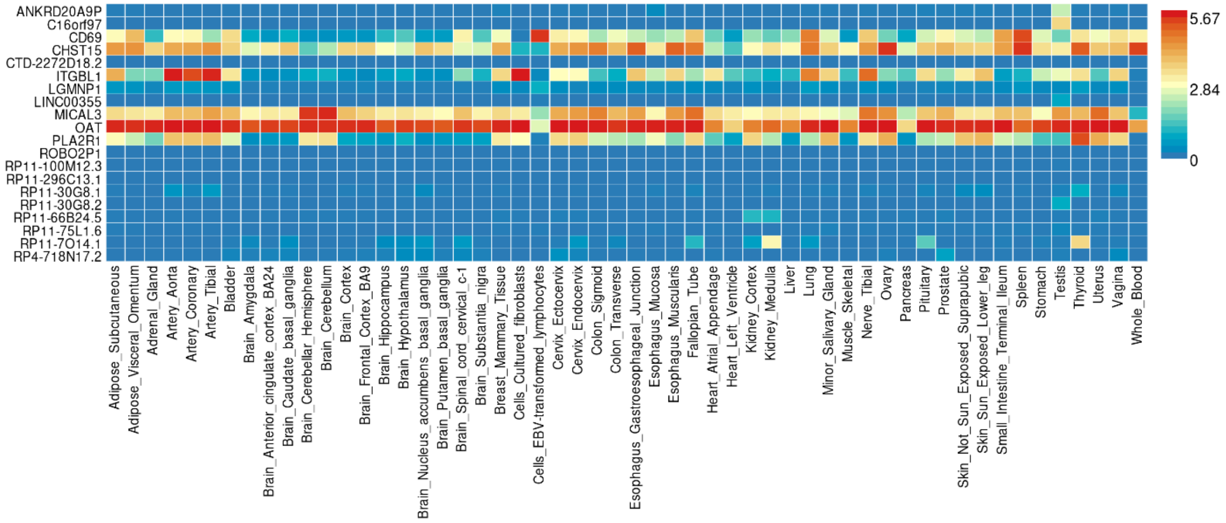

Supplementary Figure S5.

GTEx Tissue Expression Heatmap for GWAS-Significant Genes (Retrospective Cohort).

Gene expression heatmap for significant variants from the GWAS performed on the retrospective cohort using FUMA. The heatmap displays average expression levels (log2 transformed) of genes across 54 tissue types from the GTEx v8 dataset. Rows represent genes annotated to significant GWAS variants using FUMA, and columns represent tissue types arranged alphabetically. The color scale indicates expression intensity, with blue denoting low expression and red denoting high expression levels. Tissue-specific expression profiles shows that the genes associated with significant GWAS variants are expressed in tissues relevant to cancer biology, including those involved in metabolism (e.g., OAT), cell adhesion (e.g., *ITGBL1*), cytoskeletal remodeling (e.g., *MICAL3*), immune modulation (e.g., *CD69*), extracellular matrix interactions (e.g., *CHST15*), and inflammatory processes (e.g., *PLA2R1*).

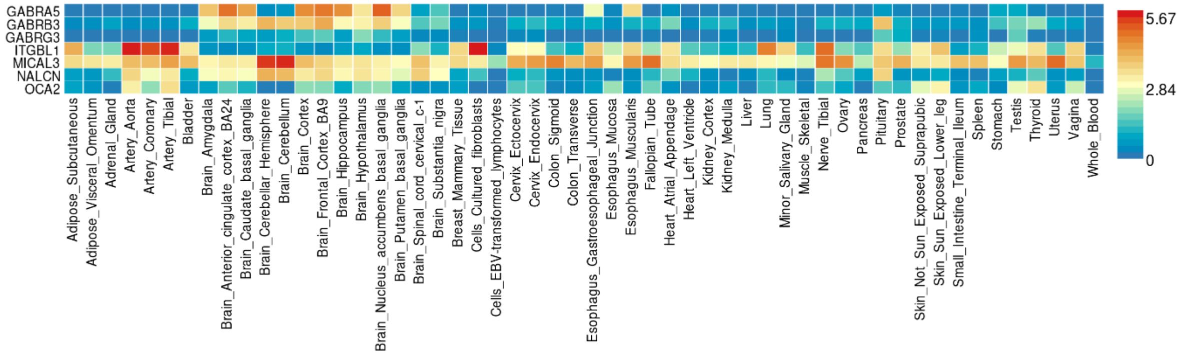

Supplementary Figure S6.
GTEx Tissue Expression Heatmap for Genes Annotated to Meta-analysis Significant SNPs.
Heatmap of tissue-specific expression levels for genes linked to variants passing the suggestive threshold ($p < 1 \times 10^{-5}$) in the meta-analysis using GTEx v8 (54 tissue types). Gene expression levels are depicted on a log-transformed scale, where red indicates higher expression, and blue indicates lower expression. Rows represent significant genes identified through FUMA annotation, while columns correspond to GTEx tissue types. Genes such as *ITGBL1* and *MICAL3* show distinct expression patterns across tissues.

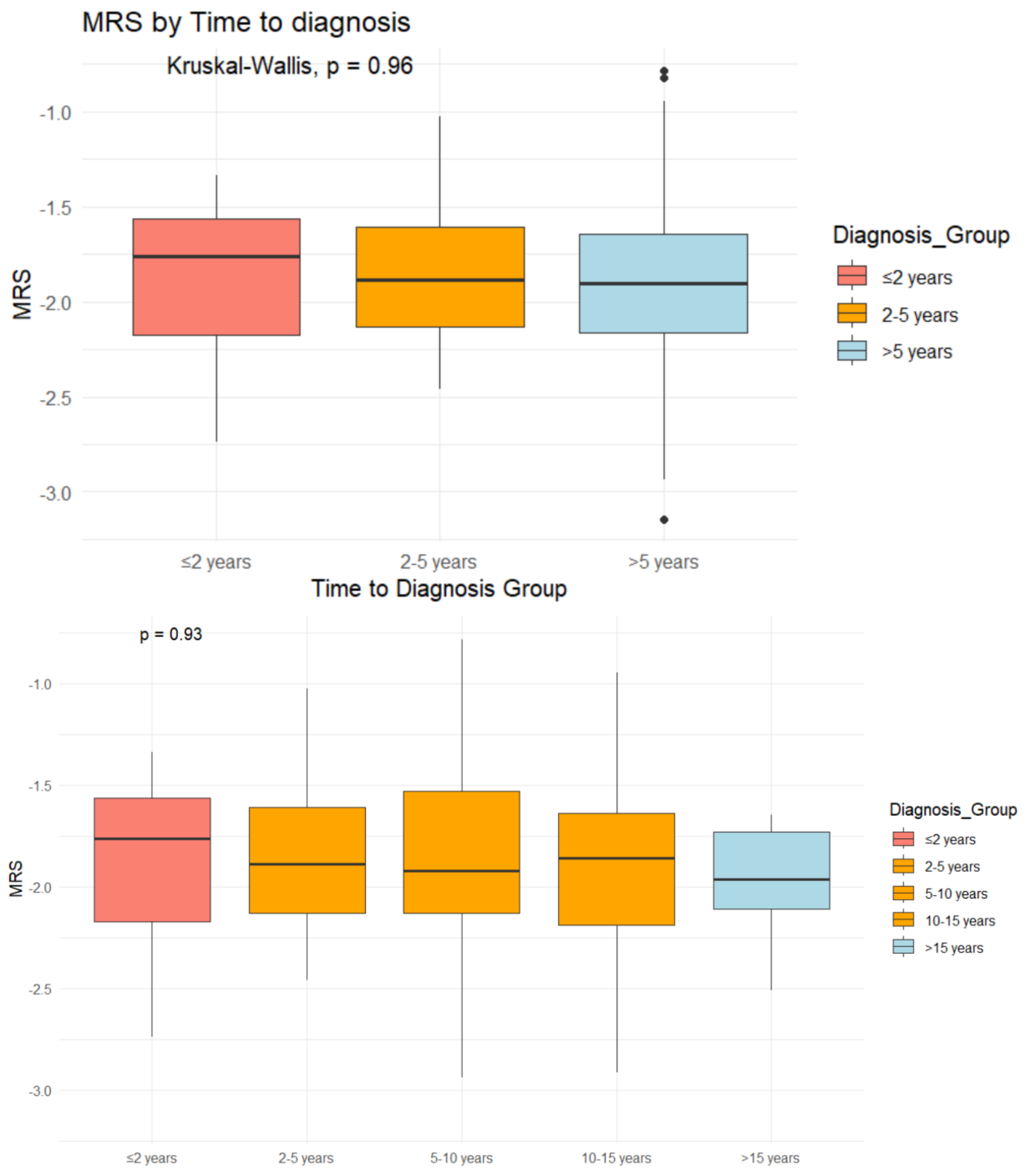

Supplementary Figure S7.
Methylation Risk Score (MRS) Distribution by Time from Sample Collection to Diagnosis.
Boxplots show MRS values stratified by time-to-diagnosis intervals in the EPIC-Meso cohort to assess whether pre-diagnostic signal intensity varies with proximity to clinical onset. The top panel illustrates MRS distributions for three groups: ≤2 years, 2-5 years, and >5 years from sample collection to diagnosis. The bottom panel provides a more granular breakdown with five intervals: ≤2 years, 2-5 years, 5-10 years, 10-15 years, and >15 years. Each box spans the interquartile range (IQR), with the horizontal line inside indicating the median MRS. Kruskal-Wallis tests yielded p = 0.96 (top panel) and p = 0.93 (bottom panel), indicating no statistically significant differences in MRS among the time-to-diagnosis groups.

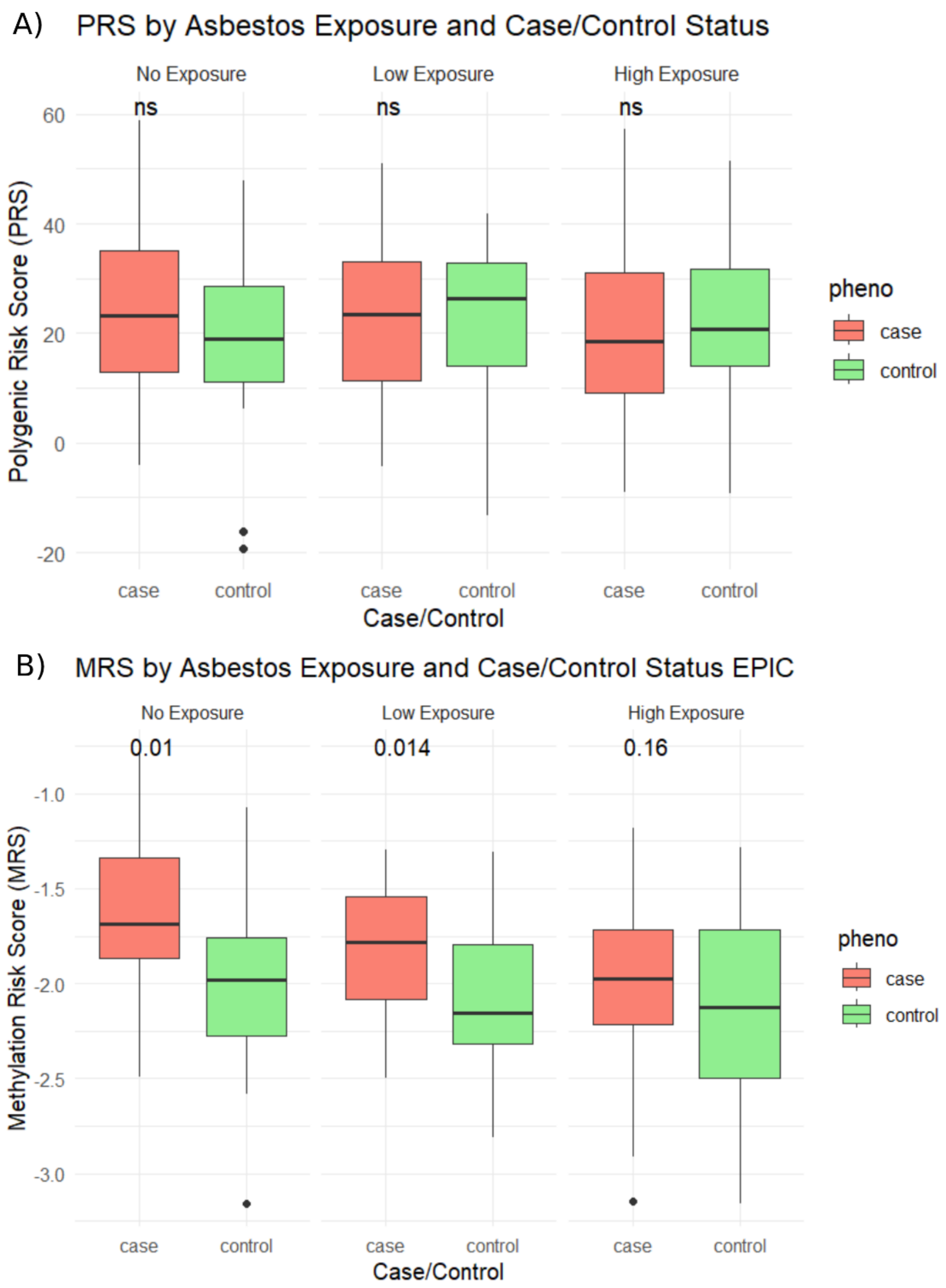

Supplementary Figure S8.

PRS and MRS Distributions by Asbestos Exposure and PM Status in the EPIC-Meso Cohort.

Box plots illustrating the distribution of **(A)** Polygenic Risk Score (PRS) and **(B)** Methylation Risk Score (MRS) by asbestos exposure levels and case/control status in the EPIC-Meso cohort. Asbestos exposure is categorized into three levels: 0 for "no exposure," 1 for "low exposure," and 2 for "high exposure." Separate boxplots are shown for cases (red) and controls (green) within each exposure category. Statistical significance between cases and controls was assessed using the Wilcoxon rank-sum test. P values are indicated, with ns = not significant ($p \geq 0.05$).

A)

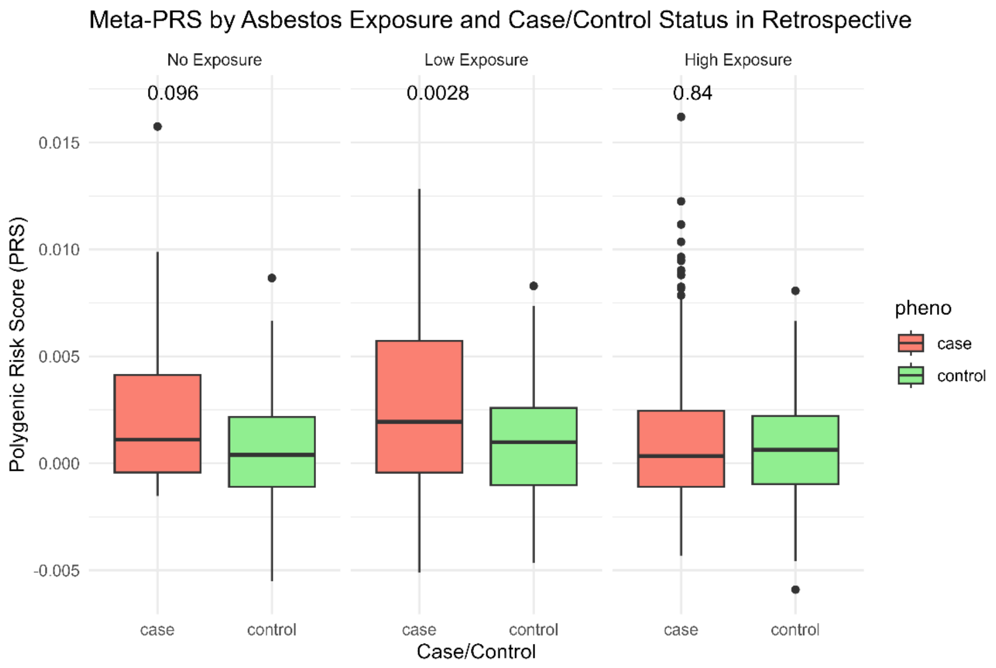

B)

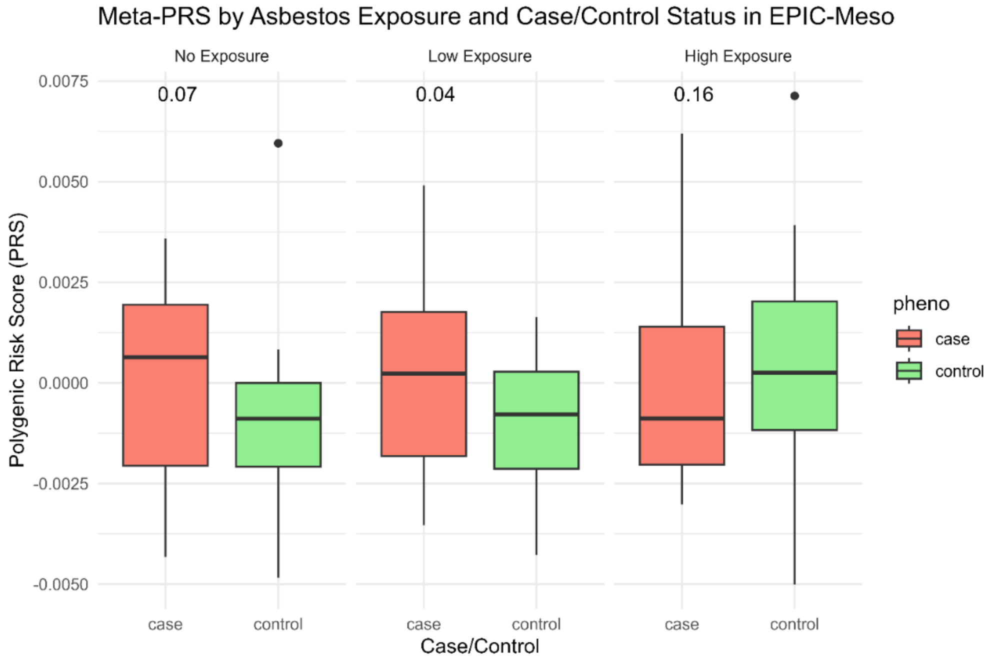

Supplementary Figure S9: Meta-PRS Distributions and performance in the full retrospective case-control study / and EPIC-Meso.

Box plots illustrating the distribution of Meta-PRS, derived from the UK Biobank-Finngen meta-analysis, by asbestos exposure levels and case/control status in **A)** the full retrospective case-control study and **B)** the EPIC-Meso cohort. Asbestos exposure is categorized into three levels: 0 for "no exposure," 1 for "low exposure," and 2 for "high exposure." Separate boxplots are shown for cases (red) and controls (green) within each exposure category. Statistical significance between cases and controls was assessed using the Wilcoxon rank-sum test. P values are indicated.

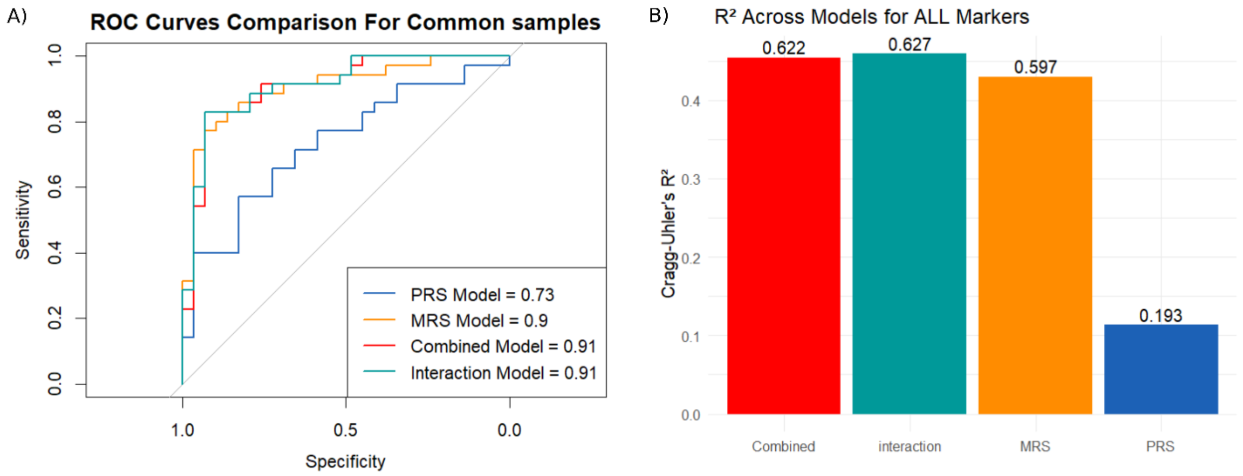

Supplementary Figure S10.

Model Performance for PRS, MRS, and Combined Models in the Retrospective Cohort subset of 64 individuals (ROC and R^2).

Receiver Operating Characteristic (ROC) curves **(A)** comparing the performance of different models in distinguishing cases from controls in the integrated retrospective cohort with 64 samples. The PRS model (blue, AUC = 0.73) uses the Polygenic Risk Score; the MRS model (orange, AUC = 0.90) uses the Methylation Risk Score; the Combined model (red, AUC = 0.91) includes both PRS and MRS as predictors; and the Interaction model (green, AUC = 0.91) includes an interaction term between PRS and MRS. Bar plot **(B)** showing Cragg-Uhler R^2 values for the PRS model, MRS model, combined model, and interaction model in the retrospective cohort.

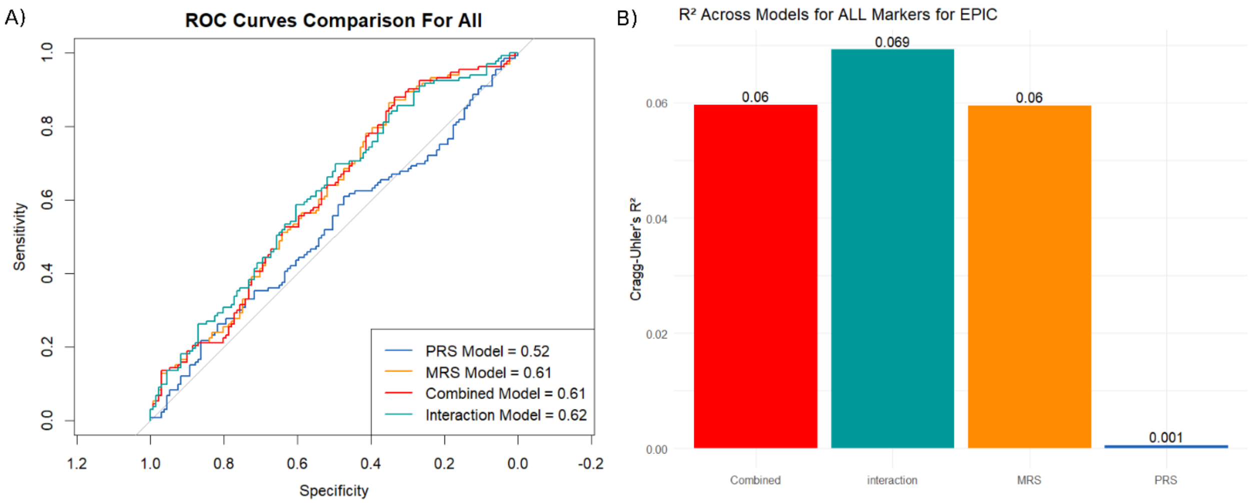

Supplementary Figure S11.

Model Performance for PRS, MRS, and Combined Models in the EPIC-Meso Cohort (ROC and R^2).

Receiver Operating Characteristic (ROC) curves **(A)** comparing the performance of different models in distinguishing cases from controls in the integrated EPIC-Meso cohort with 264 samples. The PRS model (blue, AUC = 0.73) uses the Polygenic Risk Score; the MRS model (orange, AUC = 0.90) uses the Methylation Risk Score; the Combined model (red, AUC = 0.91) includes both PRS and MRS as predictors; and the Interaction model (green, AUC = 0.91) includes an interaction term between PRS and MRS. Bar plot **(B)** showing Cragg-Uhler R^2 values for the PRS model, MRS model, combined model, and interaction model in the retrospective cohort.

Supplementary Tables:

Supplementary Table S1. Country-wise Distribution of PM Cases and Controls in the EPIC-Meso Cohort
This table shows the country-wise distribution of PM cases and controls in the EPIC-Meso cohort. Columns indicate the number of cases and controls for each participating country.

**Country**	**Case**	**Control**
D (Germany)	12	12
DK (Denmark)	40	41
ES (Spain)	8	7
FR (France)	1	1
IT (Italy)	18	17
NL (Netherlands)	5	6
SE (Sweden)	23	24
UK (United Kingdom)	27	26

Supplementary Table S2-A: Genotyping QC Filters for Retrospective Cohort Subsets (740 and 80 Samples)
Summary of filtering thresholds applied to SNP data for two sample sets (740 and 80) of retrospective cohort. MAF: Minor allele frequency, HWE: Hardy-Weinberg equilibrium, GENO: Genotyping quality, MIND: Individual missingness, Remaining: Final retained samples and genotyped SNPs after filtering.

Filter	Threshold	Sample Set 740 (Count)	Sample Set 80 (Count)
MAF	0.005	2574	0
HWE	1e-6, 1e-10	9370, 0	8870, 0
GENO	0.05	53701	4903
MIND	0.05	19 samples	0
Remaining (N samples, N variants)		721, 320724	80, 548936

Supplementary Table S2-B: Genotyping QC Summary for the EPIC-Meso Cohort
Summary of filtering thresholds applied to SNP data for EPIC-Meso samples for the genotyped data. MAF: Minor allele frequency, HWE: Hardy-Weinberg equilibrium, GENO: Genotyping quality, MIND: Individual missingness, Remaining: Final retained samples and genotyped SNPs after filtering.

Filter	Threshold	Sample Set EPIC-Meso
MAF	0.005	97782
HWE	1e-6,1e-10	8, 1
GENO	0.05	4826
MIND	0.05	0 samples
Ambiguous Sex		1 sample (F=0.69)
Heterozygosity Outliers		2 samples
Related Individuals		1 Pair, One Sample removed with low call rate
Remaining (N samples, N variants)		264 samples, 388844 variants

Supplementary Table S3: Gene Symbol and Description Mapping for Retrospective GWAS Genes Gene identifiers and descriptions for overlapping or nearby genes identified in the retrospective GWAS. Columns include the source identifier (Source Id), NCBI Entrez Gene ID (Gene Id), HGNC gene symbol (Gene Symbol), and a brief functional or descriptive annotation (Gene Description).

Source Id	NCBI (Entrez) Gene Id	Gene Symbol	Gene Description
ANKRD20A9P	284232	ANKRD20A9P	ankyrin repeat domain 20 family member A9, pseudogene [Source:HGNC Symbol;Acc:HGNC:42023]
C16orf97	388276	LINC02911	long intergenic non-protein coding RNA 2911 [Source:HGNC Symbol;Acc:HGNC:44658]
CD69	969	CD69	CD69 molecule [Source:HGNC Symbol;Acc:HGNC:1694]
CHST15	51363	CHST15	carbohydrate sulfotransferase 15 [Source:HGNC Symbol;Acc:HGNC:18137]
CTD-2272D18.2	no mapping	no mapping	no mapping
ITGBL1	9358	ITGBL1	integrin subunit beta like 1 [Source:HGNC Symbol;Acc:HGNC:6164]
LGMNP1	122199	LGMNP1	legumain pseudogene 1 [Source:HGNC Symbol;Acc:HGNC:23079]
LINC00355	144766	LINC00355	long intergenic non-protein coding RNA 355 [Source:HGNC Symbol;Acc:HGNC:27061]
MICAL3	57553	MICAL3	microtubule associated monooxygenase, calponin and LIM domain containing 3 [Source:HGNC Symbol;Acc:HGNC:24694]
OAT	4942	OAT	ornithine aminotransferase [Source:HGNC Symbol;Acc:HGNC:8091]
PLA2R1	22925	PLA2R1	phospholipase A2 receptor 1 [Source:HGNC Symbol;Acc:HGNC:9042]
ROBO2P1	106480790	ROBO2P1	roundabout guidance receptor 2 pseudogene 1 [Source:HGNC Symbol;Acc:HGNC:44386]
RP11-100M12.3	no mapping	no mapping	no mapping
RP11-296C13.1	no mapping	no mapping	no mapping
RP11-30G8.1	no mapping	no mapping	no mapping

RP11-30G8.2	no mapping	no mapping	no mapping
RP11-66B24.5	no mapping	no mapping	no mapping
RP11-75L1.6	no mapping	no mapping	no mapping
RP11-7O14.1	no mapping	no mapping	no mapping
RP4-718N17.2	no mapping	no mapping	no mapping
XXbac-B461K10.4	no mapping	no mapping	no mapping

Supplementary Table S4: Genome-wide Suggestive SNPs Identified in the EPIC-Meso GWAS: EPIC-Meso genome-wide association study (GWAS) results and mapped genes. Nearest Upstream / Downstream gene: the nearest protein coding gene within 100kb from lead SNP;
Abbreviations: CHR, chromosome; POS, base pair position based on the Genome Reference Consortium Human Build 37 (Hg19); Ref Allele, reference allele; Alt effect/alternate allele; Band, cytogenetic band region; P value, GWAS p-value.

SNP ID	CHR	POS	A1	A2	Overlapped Gene	Nearest Upstream Gene	Nearest Downstream Gene	Band	P value
rs188751136	17	73311462	G	C	-	SLC25A19	GRB2	q25.1	2.35E-07
rs62031307	16	28050907	T	C	GSG1L	-	-	p12.1	4.41E-07
rs117031010	17	73532213	G	A	LLGL2	-	-	q25.1	5.58E-07
rs117643138	17	73523643	A	G	LLGL2	-	-	q25.1	1.40E-06
rs117651797	17	73569511	C	T	LLGL2	-	-	q25.1	2.23E-06
rs4307144	6	86946640	A	G	-	NDUFA5P9	RP11-15J23.1	q14.3	3.63E-06
rs7757020	6	87043012	A	G	-	NDUFA5P9	RP11-15J23.1	q14.3	4.55E-06
rs1268403	14	21976476	T	C	METTL3	-	-	q11.2	4.89E-06
rs2162346	4	95447392	G	A	PDLIM5	-	-	q22.3	5.67E-06
rs4707260	6	86931470	C	T	-	NDUFA5P9	RP11-15J23.1	q14.3	6.22E-06
rs1577235	6	86932657	A	G	-	NDUFA5P9	RP11-15J23.1	q14.3	6.22E-06
rs7739887	6	86934692	C	A	-	NDUFA5P9	RP11-15J23.1	q14.3	6.22E-06
rs9353375	6	86937214	C	T	-	NDUFA5P9	RP11-15J23.1	q14.3	6.22E-06
rs1334648	6	86938422	G	A	-	NDUFA5P9	RP11-15J23.1	q14.3	6.22E-06
rs6454528	6	86940485	T	A	-	NDUFA5P9	RP11-15J23.1	q14.3	6.22E-06

rs72754589	1	2.27E+08	A	G	-	ITPKB	RPS27P5	q42.12	6.29E-06
rs142029448	17	73445739	G	A	KIAA0195	-	-	q25.1	6.49E-06
rs11199759	10	1.23E+08	C	T	-	RP11-95I16.2	RP11-159H3.1	q26.12	6.67E-06
rs6446661	4	4554024	A	G	STX18-AS1	-	-	p16.2	7.35E-06
rs2816579	6	87018295	A	G	-	NDUFA5P9	RP11-15J23.1	q14.3	7.38E-06
rs2798548	6	87029819	A	T	-	NDUFA5P9	RP11-15J23.1	q14.3	7.38E-06
rs2798557	6	87037330	G	C	-	NDUFA5P9	RP11-15J23.1	q14.3	7.38E-06
rs10944203	6	87044166	C	T	-	NDUFA5P9	RP11-15J23.1	q14.3	7.38E-06
rs2798538	6	86993653	T	G	-	NDUFA5P9	RP11-15J23.1	q14.3	8.59E-06
rs2324896	6	86923487	C	A	-	RP1-263J7.2	NDUFA5P9	q14.3	8.98E-06
rs2151987	6	86923994	T	A	NDUFA5P9	-	-	q14.3	8.98E-06
rs6921250	6	86925482	C	T	-	NDUFA5P9	RP11-15J23.1	q14.3	8.98E-06
rs1556626	6	86925722	G	A	-	NDUFA5P9	RP11-15J23.1	q14.3	8.98E-06
rs2151989	6	86927123	T	A	-	NDUFA5P9	RP11-15J23.1	q14.3	8.98E-06
rs1334640	6	86928017	G	A	-	NDUFA5P9	RP11-15J23.1	q14.3	8.98E-06

Supplementary Table S5: KEGG Pathway Enrichment of GWAS-Significant Genes (Retrospective Cohort)

This table reports the results of KEGG pathway enrichment analysis (via ShinyGo v8) for genes identified as significant in the retrospective GWAS. Columns include the false discovery rate (Enrichment FDR), the number of genes in each pathway (nGenes), fold enrichment values (Fold Enrichment), pathway names (Pathway), corresponding URLs, and the genes contributing to each pathway.

Enrichment FDR	nGenes	Pathway Genes	Fold Enrichment	Pathway	URL	Genes
0.026	1	20	190.675	Path:hsa00532 Glycosaminoglycan biosynthesis-chondroitin sulfate/dermatan sulfate	http://www.genome.jp/kegg-bin/show_pathway?hsa00532	CHST15
0.032	1	50	76.27	Path:hsa00330 Arginine and proline metabolism	http://www.genome.jp/kegg-bin/show_pathway?hsa00330	OAT
0.057	1	151	25.25497	Path:hsa04145 Phagosome	http://www.genome.jp/kegg-bin/show_pathway?hsa04145	PLA2R1
0.057	1	179	21.30447	Path:hsa05152 Tuberculosis	http://www.genome.jp/kegg-bin/show_pathway?hsa05152	PLA2R1

Supplementary Table S6: GO Biological Process Enrichment of GWAS-Significant Genes (Retrospective Cohort)

This table presents the Gene Ontology (GO) Biological Process enrichment analysis (via ShinyGo v8) for GWAS-significant genes from the retrospective cohort. Columns include the false discovery rate (Enrichment FDR), the number of genes (nGenes), fold enrichment values (Fold Enrichment), GO terms (Pathway), direct links to each term (URL), and the genes annotated to each term.

Enrichment FDR	nGenes	Pathway Genes	Fold Enrichment	Pathway	URL	Genes
0.04	1	8	476.6875	GO:0006561 proline biosynthetic process	http://amigo.geneontology.org/amigo/term/GO:0006561	OAT
0.04	1	8	476.6875	GO:0010519 negative regulation of phospholipase activity	http://amigo.geneontology.org/amigo/term/GO:0010519	PLA2R1
0.04	1	3	1271.167	GO:0032304 negative regulation of icosanoid secretion	http://amigo.geneontology.org/amigo/term/GO:0032304	PLA2R1
0.04	1	8	476.6875	GO:0055129 L-proline biosynthetic process	http://amigo.geneontology.org/amigo/term/GO:0055129	OAT
0.04	1	8	476.6875	GO:0090238 positive regulation of arachidonic acid secretion	http://amigo.geneontology.org/amigo/term/GO:0090238	PLA2R1
0.04	1	8	476.6875	GO:0090400 **stress-induced premature senescence**	http://amigo.geneontology.org/amigo/term/GO:0090400	PLA2R1
0.04	1	4	953.375	GO:0090403 **oxidative stress-induced premature senescence**	http://amigo.geneontology.org/amigo/term/GO:0090403	PLA2R1
0.04	1	4	953.375	GO:1900138 negative regulation of phospholipase A2 activity	http://amigo.geneontology.org/amigo/term/GO:1900138	PLA2R1
0.04	1	4	953.375	GO:1903210 glomerular visceral epithelial cell apoptotic process	http://amigo.geneontology.org/amigo/term/GO:1903210	PLA2R1
0.04	1	8	476.6875	GO:2000192 negative regulation of fatty acid transport	http://amigo.geneontology.org/amigo/term/GO:2000192	PLA2R1

0.05	1	9	423.7222	GO:0090237 regulation of arachidonic acid secretion	http://amigo.geneontology.org/amigo/term/GO:0090237	PLA2R1
0.05	1	11	346.6818	GO:0032429 regulation of phospholipase A2 activity	http://amigo.geneontology.org/amigo/term/GO:0032429	PLA2R1

Supplementary Table S7: Pathway Enrichment Analysis for Meta-Analysis Derived Suggestive SNPs ($p < 1 \times 10^{-5}$)

Pathway enrichment analysis results for suggestive variants ($p < 1 \times 10^{-5}$) identified in the meta-analysis. The analysis included 25 Entrez genes. The table details the total number of genes in each gene set (# Genes in Gene Set, K), the overlap with the two genes (# Genes in Overlap, k), the proportion of overlap (k/K), p-values, and FDR q-values. Enriched gene sets are derived from Gene Ontology (GOBP), Reactome, and WikiPathways.

Gene Set Name	# Genes in Gene Set (K)	Description	# Genes in Overlap (k)	k/K	p-value	FDR q-value
GOBP_THIAMINE_DIPHOSPHATE_METABOLIC_PROCESS	5	The chemical reactions and pathways involving thiamine diphosphate, a derivative of thiamine (vitamin B1) which acts as a coenzyme in a range of processes including the Krebs cycle. [GOC:jl, ISBN:0198506732]	2	0.4	3.31E-06	2.38E-02
REACTOME_VITAMIN_B1_THIAMIN_METABOLISM	5	Vitamin B1 (thiamin) metabolism	2	0.4	3.31E-06	2.38E-02
GOBP_THIAMINE_CONTAINING_COMPOUND_METABOLIC_PROCESS	7	The chemical reactions and pathways involving thiamine (vitamin B1), and compounds derived from it. [GOC:jl]	2	0.2857	6.94E-06	3.32E-02
WP_THIAMINE_METABOLIC_PATHWAYS	9	Thiamine metabolic pathways	2	0.2222	1.19E-05	4.27E-02

Supplementary Table S8-A: FUMA Genomic Loci Results from Meta-Analysis GWAS
Genomic loci detected by FUMA in the meta-analysis. Each row represents a distinct locus (Genomic Locus, uniqID) and includes information on the lead/reference SNP (rsID), chromosome (chr), position (pos), p-value (p), genomic boundaries (start, end), the number of mapped GWAS SNPs (nGWASSNPs), and the counts and identities of both independent significant SNPs (nIndSigSNPs, IndSigSNPs) and lead SNPs (nLeadSNPs, LeadSNPs).

Genomic Locus	uniqID	rsID	chr	pos	p	start	end	nSNPs	nGWAS SNPs	nIndSig SNPs	IndSigSNPs	nLeadS NPs	LeadSNPs
1	8:83955796:A:C	rs2122342	8	83955796	2.10E-13	83933863	83992041	17	1	1	rs2122342	1	rs2122342
2	12:9968349:G:T	rs12822999	12	9968349	2.80E-12	9968349	9968349	1	1	1	rs12822999	1	rs12822999
3	13:64718463:G:T	rs9539946	13	64718463	4.06E-12	63841610	65280676	326	1	1	rs9539946	1	rs9539946
4	13:102299158:C:G	rs1469849	13	102299158	1.89E-11	102019370	102299158	6	1	1	rs1469849	1	rs1469849
5	15:27816841:A:G	rs12900352	15	27816841	4.91E-12	26844045	28236657	74	2	2	rs12900352; rs1351034	2	rs12900352; rs1351034
6	22:18280799:A:T	rs401224	22	18280799	8.65E-09	18280799	18280799	1	1	1	rs401224	1	rs401224

Supplementary Table S8-B: FUMA Mapped Genes with Genetic Tolerance Scores and GWAS Metrics
Integrated gene mapping results from FUMA for the meta-analysis of our GWAS data. It summarizes key metrics reflecting genetic intolerance, positional mapping statistics, and the strength of GWAS associations, along with the assignment of genomic loci based on LD clustering.

ensg	symbol	chr	start	end	strand	type	entrez ID	HUGO	pLI	ncRVIS	posMap SNPs	posMapMax CADD	minGwas P	IndSigSNPs	Genomic Locus
ENSG00000102452	NALCN	13	101706130	102068843	-1	protein_coding	259232	NALCN	0.0002	0.5571	2	4.58	NA	rs1469849	4
ENSG00000198542	ITGBL1	13	102104966	102375456	1	protein_coding	9358	ITGBL1	0.1709	0.1877	4	2.88	1.89E-11	rs1469849	4
ENSG00000166206	GABRB3	15	26788693	27184686	-1	protein_coding	2562	GABRB3	0.9971	-1.8900	3	0.42	NA	rs12900352	5
ENSG00000186297	GABRA5	15	27111510	27194354	1	protein_coding	2558	GABRA5	0.9515	-1.3954	1	0.11	NA	rs12900352	5
ENSG00000182256	GABRG3	15	27216429	27778373	1	protein_coding	2567	GABRG3	0.9937	NA	4	8.86	NA	rs12900352	5
ENSG00000104044	OCA2	15	28000021	28344504	-1	protein_coding	4948	OCA2	7.68E-12	NA	2	0.66	NA	rs12900352	5
ENSG00000243156	MICAL3	22	18270415	18507325	-1	protein_coding	57553	MICAL3	0.999998	-0.7222	1	0.37	8.65E-09	rs401224	6

Supplementary Table S9: Gene Symbol and Description Mapping for the 54 Differentially Methylated Genes- Gene identifiers and descriptions for overlapping or nearby genes identified in differential methylation analysis in the retrospective case-control study. Columns include the source identifier (Source Id), NCBI Entrez Gene ID (Gene Id), HGNC gene symbol (Gene Symbol), and a brief functional or descriptive annotation (Gene Description).

Source Id	NCBI (Entrez) Gene Id	Gene Symbol	Gene Description
ACSF3	197322	ACSF3	acyl-CoA synthetase family member 3 [Source:HGNC Symbol
ATXN7	6314	ATXN7	ataxin 7 [Source:HGNC Symbol
C18orf1	753	LDLRAD4	low density lipoprotein receptor class A domain containing 4 [Source:HGNC Symbol
CDC25B	994	CDC25B	cell division cycle 25B [Source:HGNC Symbol
CLEC4A	50856	CLEC4A	C-type lectin domain family 4 member A [Source:HGNC Symbol
CLSTN1	22883	CLSTN1	calsyntenin 1 [Source:HGNC Symbol
CPNE3	8895	CPNE3	copine 3 [Source:HGNC Symbol
CXCR6	10663	CXCR6	C-X-C motif chemokine receptor 6 [Source:HGNC Symbol
DCAF5	8816	DCAF5	DDB1 and CUL4 associated factor 5 [Source:HGNC Symbol
EP400	57634	EP400	E1A binding protein p400 [Source:HGNC Symbol
ESPNL	339768	ESPNL	espin like [Source:HGNC Symbol
FAM120B	84498	FAM120B	family with sequence similarity 120 member B [Source:HGNC Symbol
FER1L5	90342	FER1L5	fer-1 like family member 5 [Source:HGNC Symbol
FKBP5	2289	FKBP5	FKBP prolyl isomerase 5 [Source:HGNC Symbol
FLJ42289	388182	SPATA41	spermatogenesis associated 41 [Source:HGNC Symbol
FNDC3B	64778	FNDC3B	fibronectin type III domain containing 3B [Source:HGNC Symbol
FOXK1	221937	FOXK1	forkhead box K1 [Source:HGNC Symbol
FYCO1	79443	FYCO1	FYVE and coiled-coil domain autophagy adaptor 1 [Source:HGNC Symbol
HHLA2	11148	HHLA2	HHLA2 member of B7 family [Source:HGNC Symbol
HPS3	84343	HPS3	HPS3 biogenesis of lysosomal organelles complex 2 subunit 1 [Source:HGNC Symbol
ITPR1	3708	ITPR1	inositol 1,4,5-trisphosphate receptor type 1 [Source:HGNC Symbol
KCNQ1	3784	KCNQ1	potassium voltage-gated channel subfamily Q member 1 [Source:HGNC Symbol
KDM4A	9682	KDM4A	lysine demethylase 4A [Source:HGNC Symbol
LIME1	54923	LIME1	Lck interacting transmembrane adaptor 1 [Source:HGNC Symbol
LPGAT1	9926	LPGAT1	lysophosphatidylglycerol acyltransferase 1 [Source:HGNC Symbol

MAML3	55534	MAML3	mastermind like transcriptional coactivator 3 [Source:HGNC Symbol
MNAT1	4331	MNAT1	MNAT1 component of CDK activating kinase [Source:HGNC Symbol
MORC2	22880	MORC2	MORC family CW-type zinc finger 2 [Source:HGNC Symbol
MSH6	2956	MSH6	mutS homolog 6 [Source:HGNC Symbol
MYOZ3	91977	MYOZ3	myozenin 3 [Source:HGNC Symbol
NRXN2	9379	NRXN2	neurexin 2 [Source:HGNC Symbol
PCCA	5095	PCCA	propionyl-CoA carboxylase subunit alpha [Source:HGNC Symbol
PHLPP1	23239	PHLPP1	PH domain and leucine rich repeat protein phosphatase 1 [Source:HGNC Symbol
PNKD	25953	PNKD	PNKD metallo-beta-lactamase domain containing [Source:HGNC Symbol
PPARG	5468	PPARG	peroxisome proliferator activated receptor gamma [Source:HGNC Symbol
RALGPS1	9649	RALGPS1	Ral GEF with PH domain and SH3 binding motif 1 [Source:HGNC Symbol
SEC22A	26984	SEC22A	SEC22 homolog A, vesicle trafficking protein [Source:HGNC Symbol
SGPL1	8879	SGPL1	sphingosine-1-phosphate lyase 1 [Source:HGNC Symbol
SLC43A2	124935	SLC43A2	solute carrier family 43 member 2 [Source:HGNC Symbol
TAF4	6874	TAF4	TATA-box binding protein associated factor 4 [Source:HGNC Symbol
TAP1	6890	TAP1	transporter 1, ATP binding cassette subfamily B member [Source:HGNC Symbol
TBC1D4	9882	TBC1D4	TBC1 domain family member 4 [Source:HGNC Symbol
TCEB1	6921	ELOC	elongin C [Source:HGNC Symbol
THADA	63892	THADA	THADA armadillo repeat containing [Source:HGNC Symbol
TMEM182	130827	TMEM182	transmembrane protein 182 [Source:HGNC Symbol
TPD52	7163	TPD52	tumor protein D52 [Source:HGNC Symbol
TRIB1	10221	TRIB1	tribbles pseudokinase 1 [Source:HGNC Symbol
TRIM27	5987	TRIM27	tripartite motif containing 27 [Source:HGNC Symbol
TSLP	85480	TSLP	thymic stromal lymphopoietin [Source:HGNC Symbol
VPS37C	55048	VPS37C	VPS37C subunit of ESCRT-I [Source:HGNC Symbol
VRK2	7444	VRK2	VRK serine/threonine kinase 2 [Source:HGNC Symbol
ZDHHC2	51201	ZDHHC2	zinc finger DHHC-type palmitoyltransferase 2 [Source:HGNC Symbol
ZFP36L1	677	ZFP36L1	ZFP36 ring finger protein like 1 [Source:HGNC Symbol

Supplementary Table S10-A: PRS Distributions by Asbestos Exposure Group in the Retrospective Test Set:

This table presents the Polygenic Risk Score (PRS) distributions for cases and controls across no exposure, low exposure, and high exposure groups in the retrospective cohort. Columns include Exposure (asbestos exposure level: 0 = no exposure, 1 = low exposure, 2 = high exposure), Pheno (case/control status), N (number of participants), Mean (average score), SD (standard deviation), SE (standard error), and CI_lower and CI_upper (95% confidence interval for the mean).

Exposure	Pheno	N	Mean	SD	SE	CI_lower	CI_upper
0	case	11	8.89	20.6	6.21	-4.95	22.7
0	control	52	-4.02	16.9	2.34	-8.72	0.676
1	case	84	10.3	18	1.97	6.35	14.2
1	control	73	-1.21	12.4	1.45	-4.11	1.69
2	case	99	2.68	17.2	1.73	-0.75	6.11
2	control	56	0.0911	15.2	2.03	-3.98	4.17

Supplementary Table S10-B: MRS Distributions by Asbestos Exposure Group in the Retrospective Test Set:

This table presents the Methylation Risk Score (MRS) distributions for cases and controls across no exposure, low exposure, and high exposure groups in the retrospective cohort. Columns include Exposure (asbestos exposure level: 0 = no exposure, 1 = low exposure, 2 = high exposure), Pheno (case/control status), N (number of participants), Mean (average score), SD (standard deviation), SE (standard error), and CI_lower and CI_upper (95% confidence interval for the mean).

Exposure	Pheno	N	Mean	SD	SE	CI_lower	CI_upper
0	case	2	-1.98	0.726	0.513	-8.5	4.54
0	control	8	-2.39	0.604	0.213	-2.9	-1.89
1	case	41	-1.27	0.668	0.104	-1.49	-1.06
1	control	41	-2.22	0.712	0.111	-2.44	-2
2	case	37	-1.33	0.653	0.107	-1.55	-1.12
2	control	21	-2.27	0.88	0.192	-2.67	-1.87

Supplementary Table S11-A: PRS Distributions by Asbestos Exposure Group in EPIC-Meso Cohort: This table presents the Polygenic Risk Score (PRS) distributions for cases and controls across no exposure, low exposure, and high exposure groups in the EPIC-Meso cohort. Columns include Exposure (asbestos exposure level: 0 = no exposure, 1 = low exposure, 2 = high exposure), Pheno (case/control status), N (number of participants), Mean (average score), SD (standard deviation), SE (standard error), and CI_lower and CI_upper (95% confidence interval for the mean).

Exposure	Pheno	N	Mean	SD	SE	CI_lower	CI_upper
0	case	20	22.7	16.8	3.75	14.8	30.5
0	control	20	18.7	17.1	3.83	10.6	26.7
1	case	28	23.5	14.2	2.68	18	29
1	control	26	22.9	13.8	2.7	17.3	28.5
2	case	42	21.3	15.5	2.39	16.5	26.1
2	control	43	21.5	13.9	2.12	17.2	25.8

Supplementary Table S11-B: MRS Distributions by Asbestos Exposure Group in EPIC-Meso Cohort: This table presents the Methylation Risk Score (MRS) distributions for cases and controls across no exposure, low exposure, and high exposure groups in the EPIC-Meso cohort. Columns include Exposure (asbestos exposure level: 0 = no exposure, 1 = low exposure, 2 = high exposure), Pheno (case/control status), N (number of participants), Mean (average score), SD (standard deviation), SE (standard error), and CI_lower and CI_upper (95% confidence interval for the mean).

Exposure	Pheno	N	Mean	SD	SE	CI_lower	CI_upper
0	case	21	-1.65	0.446	0.0974	-1.85	-1.45
0	control	22	-2.01	0.463	0.0987	-2.21	-1.8
1	case	28	-1.84	0.36	0.068	-1.98	-1.7
1	control	27	-2.09	0.378	0.0728	-2.24	-1.94
2	case	43	-1.98	0.396	0.0604	-2.11	-1.86
2	control	43	-2.14	0.464	0.0708	-2.29	-2

Supplementary Table S12-A: Meta-PRS Distributions by Asbestos Exposure Group in the full Retrospective Case-Control Study:

This table presents the Meta-PRS distributions, derived from the UK Biobank-Finngen meta-analysis, for cases and controls across no exposure, low exposure, and high exposure groups in the full retrospective case-control study. Columns include Exposure (asbestos exposure level: 0 = no exposure, 1 = low exposure, 2 = high exposure), Pheno (case/control status), N (number of participants), Mean (average score), SD (standard deviation), SE (standard error), and CI_lower and CI_upper (95% confidence interval for the mean).

Exposure	Pheno	N	Mean	SD	SE	CI_lower	CI_upper
0	case	17	0.00274	0.00447	0.00108	0.000441	0.00503
0	control	107	0.000617	0.0025	0.000241	0.000139	0.0011
1	case	178	0.00271	0.00421	0.000316	0.00209	0.00333
1	control	153	0.00105	0.00257	0.000208	0.000634	0.00146
2	case	192	0.00119	0.00355	0.000256	0.000681	0.00169
2	control	102	0.000698	0.00244	0.000242	0.000218	0.00118

Supplementary Table S12-B: Meta-PRS Distributions by Asbestos Exposure Group in the EPIC-Meso Study:

This table presents the Meta-PRS distributions, derived from the UK Biobank-Finngen meta-analysis, for cases and controls across no exposure, low exposure, and high exposure groups in the EPIC-Meso study. Columns include Exposure (asbestos exposure level: 0 = no exposure, 1 = low exposure, 2 = high exposure), Pheno (case/control status), N (number of participants), Mean (average score), SD (standard deviation), SE (standard error), and CI_lower and CI_upper (95% confidence interval for the mean).

Exposure	Pheno	N	Mean	SD	SE	CI_lower	CI_upper
0	case	21	0.000129	0.00232	0.000507	-0.00093	0.00119
0	control	22	-0.00098	0.00222	0.000474	-0.00196	6.51E-06
1	case	28	0.000187	0.00235	0.000444	-0.00072	0.0011
1	control	27	-0.00108	0.00174	0.000335	-0.00177	-0.00039
2	case	43	-0.00016	0.00238	0.000364	-0.00089	0.000576
2	control	43	0.000339	0.00237	0.000362	-0.00039	0.00107

Supplementary Table S13: SNP × Asbestos Exposure Interaction Results from Logistic Regression

This table presents the results of a logistic regression analysis evaluating the association between selected SNPs and PM risk, adjusted for asbestos exposure, age, sex, and principal components (PCs). The SNP OR (95%CI) column reports the odds ratio (OR) and 95% confidence interval (CI) for each SNP. The Exposure OR (95%CI) column presents the OR for asbestos exposure. The Interaction OR (95%CI) column estimates the SNP-exposure interaction effect. The three p- value columns: SNP p- value, Exposure p- value and Interaction p- value indicate statistical significance for each term

SNP ID	SNP OR (95%CI)	SNP P value	Exposure OR (95%CI)	Exposure P value	Interaction OR (95%CI)	Interaction P value
rs1877196	0.49 (0.2-1.18)	0.112	5.38 (2.35-12.29)	**6.68E-05**	2.09 (0.84-5.2)	0.112
rs2876969	0 (0-Inf)	0.995	8.34 (4.78-14.55)	**8.65E-14**	159548484914827 (0-Inf)	0.990
**rs2122342**	**0.24 (0.1-0.62)**	**0.003**	4.13 (0.79-21.7)	0.094	1.62 (0.61-4.3)	0.337
**rs2459219**	**11.91 (1.44-98.72)**	**0.022**	10.07 (5.53-18.34)	**4.46E-14**	1538428.1 (0-Inf)	0.976
**rs12822999**	**14.94 (1.34-167.01)**	**0.028**	9.79 (5.38-17.82)	**8.57E-14**	0.54 (0.04-7.35)	0.645
rs9539946	0.62 (0.19-2.06)	0.433	9.93 (4.87-20.24)	**2.60E-10**	0.94 (0.27-3.23)	0.917
rs1469849	0.61 (0.18-2.03)	0.420	9.95 (4.88-20.3)	**2.62E-10**	0.94 (0.27-3.23)	0.918
rs113946246	0.5 (0.13-1.86)	0.298	9.53 (4.79-18.96)	**1.32E-10**	1.17 (0.3-4.52)	0.815
rs2490010	0.54 (0.15-2.02)	0.362	9.91 (4.99-19.67)	**5.56E-11**	1.06 (0.28-4.07)	0.933
rs67206049	0.52 (0.14-1.96)	0.337	9.77 (4.92-19.41)	**7.60E-11**	1.11 (0.29-4.27)	0.879
rs2440012	8548290.29 (0-Inf)	0.980	9.01 (5.02-16.14)	**1.56E-13**	0 (0-Inf)	0.984
rs1838126	780055922.43 (0-Inf)	0.996	9.65 (5.31-17.57)	**1.13E-13**	0.04 (0-Inf)	0.999
rs12912424	148492206.82 (0-Inf)	0.996	8.61 (4.87-15.23)	**1.26E-13**	0.09 (0-Inf)	1.000
rs12900352	8498467.68 (0-Inf)	0.980	9.16 (5.11-16.44)	**1.06E-13**	0 (0-Inf)	0.983
**rs1351034**	**9.21 (1.37-62.05)**	**0.022**	9.64 (5.27-17.61)	**1.76E-13**	1.01 (0.12-8.65)	0.992
rs1079859	286057912.75 (0-Inf)	0.994	9.24 (5.07-16.84)	**3.76E-13**	0.07 (0-Inf)	0.999
rs401224	3.42 (0.72-16.2)	0.122	9.43 (5.25-16.96)	**6.38E-14**	1.49 (0.26-8.61)	0.655

Supplementary Table S14: Stratified SNP Associations by Asbestos Exposure Status
This table shows the association of SNPs with pleural mesothelioma risk stratified by asbestos exposure. SNP OR (95%CI) Exposed and SNP OR (95%CI) Unexposed represents OR (with 95% CI) for SNP effects in exposed and unexposed groups, respectively. SNP P Exposed and SNP P Unexposed indicate statistical significance for each stratum.

SNP ID	SNP OR (95%CI) Exposed	SNP P Exposed	SNP OR (95%CI) Unexposed	SNP P Unexposed
rs1079859	18795156.75 (0-Inf)	0.9743	92207320.33 (0-Inf)	0.9907
**rs113946246**	0.58 (0.43-0.79)	**0.0004**	0.62 (0.15-2.53)	0.5022
**rs12822999**	8.07 (3.00-21.72)	**3.49e-050**	19.73 (1.68-233.15)	**0.0179**
**rs12900352**	15.67 (3.90-63.22)	**0.0001**	138922695.50 (0-Inf)	0.9910
rs12912424	14163353.22 (0-Inf)	0.9696	20537631.03 (0-Inf)	0.9908
**rs1351034**	9.57(3.53-25.95)	**9.13e-06**	13.03(1.23-138.13)	**0.0331**
**rs1469849**	0.58 (0.42-0.77)	**0.0002**	0.76 (0.20-2.84)	0.6849
rs1838126	29868763.04 (0-Inf)	0.9788	132462298.09 (0-Inf)	0.9891
**rs1877196**	1.0361 (0.80-1.33)	0.7778	0.40 (0.15-1.09)	**0.0745**
**rs2122342**	0.3947 (0.30-0.53)	**5.00e-10**	0.21 (0.07-0.59)	**0.0034**
**rs2440012**	27.14 (3.88-189.87)	**0.0009**	138922695.50 (0-Inf)	0.9910
rs2459219	17986589.23 (0-Inf)	0.9719	12.88 (1.37-121.39)	**0.0256**
**rs2490010**	0.58 (0.4274-0.7731)	**0.0002**	0.64 (0.16-2.65)	0.5422
rs2876969	19271530.68 (0-Inf)	0.9761	0 (0-Inf)	0.9930
**rs401224**	5.03 (2.25-11.24)	**8.50e-05**	9.71 (0.89-105.65)	**0.0618**
**rs67206049**	0.58 (0.43-0.78)	**0.0003**	0.63 (0.15-2.59)	0.5235
**rs9539946**	0.58 (0.43-0.78)	**0.0003**	0.78 (0.20-2.91)	0.7079

Supplementary Table S15: Significant RERI Results for SNP × Exposure Interactions:
This table presents the Relative Excess Risk due to Interaction (RERI) for SNP-exposure interactions in pleural mesothelioma risk. OR values represent odds ratios for different exposure-genotype combinations: OR_10 (exposed, reference genotype), OR_01 (unexposed, risk allele present), and OR_11 (both exposed and carrying the risk allele).

SNP ID	OR_10	OR_01	OR_11	RERI	CI_lower	CI_upper
rs9539946	9.93	0.62	0.94	-8.61	-15.82	-1.41
rs1469849	9.95	0.61	0.94	-8.62	-15.85	-1.40
rs113946246	9.53	0.50	1.17	-7.86	-14.63	-1.08
rs2490010	9.91	0.54	1.06	-8.39	-15.37	-1.41
rs67206049	9.77	0.52	1.11	-8.18	-15.09	-1.28
rs401224	9.43	3.42	1.49	-10.36	-18.46	-2.25
